# Live cell screening platform identifies PPARδ as a regulator of cardiomyocyte proliferation and cardiac repair

**DOI:** 10.1038/cr.2017.84

**Published:** 2017-06-16

**Authors:** Ajit Magadum, Yishu Ding, Lan He, Teayoun Kim, Mohankrishna Dalvoy Vasudevarao, Qinqiang Long, Kevin Yang, Nadeera Wickramasinghe, Harsha V Renikunta, Nicole Dubois, Gilbert Weidinger, Qinglin Yang, Felix B Engel

**Affiliations:** 1 Department of Cardiac Development and Remodelling, Max-Planck-Institute for Heart and Lung Research, Parkstrasse 1, Bad Nauheim 61231, Germany; 2 Department of Cardiology, Icahn School of Medicine at Mount Sinai Hospital, One Gustave L. Levy Place, Box 1030, New York, NY 10029, USA; 3 Department of Nutrition Sciences, University of Alabama at Birmingham, 1675 University Blvd, Birmingham, AL 35294-3360, USA; 4 Institute of Biochemistry and Molecular Biology, Ulm University, Albert-Einstein-Allee 11, Ulm 89081, Germany; 5 Division of Cardiology, Department of Internal Medicine, Tongji Hospital, Tongji Medical College, Huazhong University of Science and Technology, 1095 Jiefang Ave, Wuhan, Hubei 430030, China; 6 Department for Cell, Developmental and Regenerative Biology, Icahn School of Medicine at Mount Sinai, 1470 Madison Avenue, Box 1040, New York, NY 10029, USA; 7 Department of Nephropathology, Experimental Renal and Cardiovascular Research, Institute of Pathology, Friedrich-Alexander-Universität Erlangen-Nürnberg, Schwabachanlage 12, Erlangen 91054, Germany; 8 Muscle Research Center Erlangen (MURCE)

**Keywords:** cardiac repair, cardiomyocyte proliferation, screening, carbacyclin, PPARδ, GSK3β, Tbx20

## Abstract

Zebrafish can efficiently regenerate their heart through cardiomyocyte proliferation. In contrast, mammalian cardiomyocytes stop proliferating shortly after birth, limiting the regenerative capacity of the postnatal mammalian heart. Therefore, if the endogenous potential of postnatal cardiomyocyte proliferation could be enhanced, it could offer a promising future therapy for heart failure patients. Here, we set out to systematically identify small molecules triggering postnatal cardiomyocyte proliferation. By screening chemical compound libraries utilizing a Fucci-based system for assessing cell cycle stages, we identified carbacyclin as an inducer of postnatal cardiomyocyte proliferation. *In vitro*, carbacyclin induced proliferation of neonatal and adult mononuclear rat cardiomyocytes via a peroxisome proliferator-activated receptor δ (PPARδ)/PDK1/p308Akt/GSK3β/β-catenin pathway. Inhibition of PPARδ reduced cardiomyocyte proliferation during zebrafish heart regeneration. Notably, inducible cardiomyocyte-specific overexpression of constitutively active PPARδ as well as treatment with PPARδ agonist after myocardial infarction in mice induced cell cycle progression in cardiomyocytes, reduced scarring, and improved cardiac function. Collectively, we established a cardiomyocyte proliferation screening system and present a new drugable target with promise for the treatment of cardiac pathologies caused by cardiomyocyte loss.

## Introduction

It has been projected that the future socio-economic burden of heart disease will further increase due to aging populations in high-income countries and to the increasing incidence of cardiac risk factors such as hypertension, obesity and diabetes in low-income countries. Thus, it is important to identify strategies for repairing the heart because conventional treatment regimens fail to prevent cardiomyocyte loss and cannot reverse cardiac damage^1^.

Zebrafish can regenerate their adult heart through cardiomyocyte proliferation^2^. Yet, in the mammalian heart, regeneration is limited to newborns because cardiomyocyte proliferation rapidly ceases after birth^3^. Why mammalian cardiomyocytes stop proliferating after birth and whether this block is permanent is unclear. Recently, several explanations have been put forward including gene silencing^4^, DNA damage^5^ and centrosome disassembly^6^. While these data suggest that the cell cycle arrest in postnatal cardiomyocytes is permanent, several publications have provided evidence that induction of cardiomyocyte proliferation in the adult mammalian heart improves cardiac function^7,8,9^. Thus, there might be a subpopulation of mammalian cardiomyocytes, as previously suggested^10,11^, that behaves like adult zebrafish cardiomyocytes. Importantly, therapy for heart failure is elusive and even in zebrafish, the mechanism of cardiac regeneration is still poorly understood.

Here, we set out to systematically identify small molecules triggering proliferation of neonatal mononuclear rat cardiomyocytes on the assumption that signaling pathways required for proliferation of zebrafish cardiomyocytes are conserved and would act to promote proliferation of a presumptive mononuclear adult cardiomyocyte subpopulation in mammals. For this purpose we established a simple, fast, affordable and efficient cardiomyocyte-specific screening system based on the Fucci system in which cells at specific phases of the cell cycle express phase-specific fluorescent markers^12^. Our screen and subsequent animal experiments identified the peroxisome proliferator-activated receptor delta (PPARδ, also known as PPARβ), a nuclear hormone receptor and transcription factor^13^, as a promising target for therapeutic induction of cardiomyocyte proliferation. PPARδ has previously been associated with a variety of proliferative diseases that include hyperproliferative skin disorders and cancer as well as with proliferation and cell survival of several cell types^14,15^. PPARδ has also been linked to wound healing and tissue repair due to cell proliferation in mammalian skin, liver, muscle and cornea^16,17,18,19^. The roles of PPARδ in transcriptional control of physiological and pathological processes have been recently reviewed^13^.

PPARδ is highly expressed in the embryonic as well as adult heart and known as a powerful regulator of fatty acid catabolism and energy homeostasis^20,21^. PPARδ knockout leads to embryonic lethality and growth retardation in the surviving mice^22,23^. The role of PPARδ has previously also been investigated in different heart diseases^24^. For instance, we have shown that cardiomyocyte-restricted PPARδ deletion in mice perturbs myocardial fatty acid oxidation causing cardiac dysfunction leading to hypertrophy, fibrosis and lipotoxic cardiomyopathy^25^. In contrast, cardiac-specific overexpression of PPARδ is protective to ischemia/reperfusion (I/R) injury as well as TAC-induced pressure overload^26,27^. However, there are no reports on the role of PPARδ in cardiomyocyte proliferation *in vitro* or *in vivo*. Here we show that carbacyclin induces cardiomyocyte proliferation via a PPARδ/PDK1/p308Akt/GSK3β/β-catenin-pathway and that activated PPARδ signaling after myocardial infarction (MI) induces cardiomyocyte cell cycle activity and improves scarring as well as cardiac function. Moreover, inhibition of PPARδ reduces cardiomyocyte proliferation during zebrafish heart regeneration.

## Results

### Screening for small molecules promoting cardiomyocyte proliferation *in vitro*

The so-called Fucci system, developed to monitor cell cycle progression, makes use of the fact that Cdt1 (a marker of G1/G0 phase) and Geminin (a marker of S, G2 and M phases) are subject to cell cycle-dependent proteolysis^12^. Thus, the Fucci system can be used to screen for potential inducers of cell cycle re-entry through either loss of the Cdt1 signal or gain of the Geminin signal (Supplementary information, Figure S1A).

We first considered monitoring the loss of Cdt1 signal. Adenovirus (Ad)-mediated transfection of primary postnatal Day 3 (P3) rat cardiomyocytes with a non-functional hCdt1 deletion mutant fused to mCherry (mCherry-hCdt1(30/120)) under the control of the cardiomyocyte-specific α-MHC promoter (Ad-mCherry-hCdt1(30/120) resulted in the expression of mCherry-hCdt1(30/120) in > 90% of neonatal cardiomyocytes based on mCherry staining and counterstaining against cardiomyocyte-specific Troponin I (Supplementary information, Figure S1B-S1D). However, a markedly lower number of mCherry-hCdt1(30/120)-positive cardiomyocytes were detected in observations of live cells (Supplementary information, Figure S1E). In addition, screening based on the decrease in the number of mCherry-hCdt1(30/120)-positive cells appears to be error prone due to the unavoidable presence of non-myocytes; drug-induced non-myocyte proliferation will result in a “false loss of signal” (Supplementary information, Figure S1F). Thus, we did not further pursue loss of Cdt1 as a readout for induction of cardiomyocyte proliferation.

**Figure 1:**
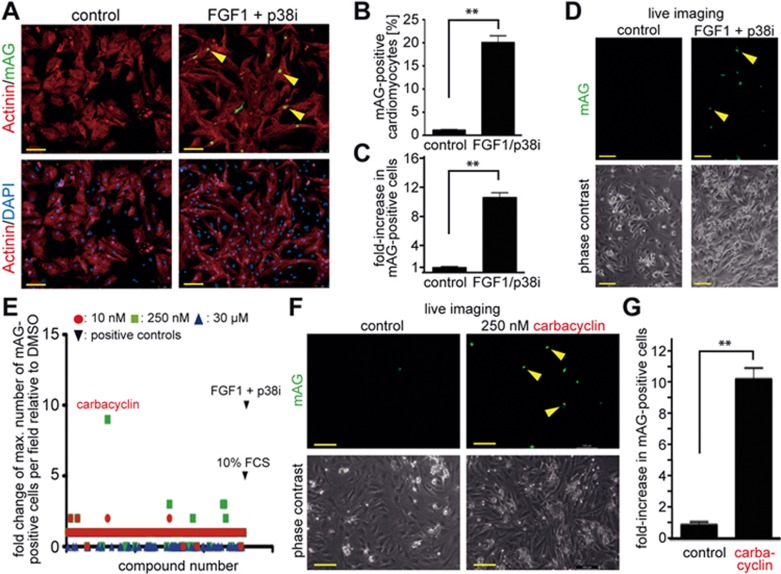
Chemical library screen identifies carbacyclin as a potential inducer of cardiomyocyte proliferation. **(A)** Representative examples of Ad-mAG-hGem(1/110) infected postnatal cardiomyocyte cultures after serum starvation (control) or stimulation with FGF1/p38i stained for mAG expression (green), cardiomyocyte-specific Actinin (red), and DNA (DAPI, blue). Yellow arrowheads, mAG-positive cardiomyocyte. **(B)** Quantitative analysis of **A** (*n* = 6). **(C, D)** Quantitative analysis (*n* = 6) and representative live images of control or FGF1 + p38i-stimulated cardiomyocyte cultures infected with Ad-mAG-hGem(1/110) (green). **(E)** Quantitative analysis of the screen of a nuclear receptor ligand library and an epigenetics screening library. **(F, G)** Quantitative analysis (*n* = 6) and representative live images of control- or carbacyclin-treated neonatal cardiomyocyte cultures infected with Ad-mAG-hGem(1/110) (green). Yellow arrowheads: mAG-positive cells. ^**^P < 0.01. Scale bar = 100 μm.

We then turned to monitoring Geminin expression. Immunofluorescence analyses revealed that adenoviral overexpression of a non-functional human Geminin deletion mutant fused to a monomeric version of Azami Green (mAG-hGem(1/110)) in P3 rat cardiomyocytes *in vitro* under the control of the α-MHC promoter resulted in the expression of mAG-hGem(1/110) in < 1.5% of cardiomyocytes (Figure 1A and 1B). This suggests that overexpressed mAG-hGem(1/110) is actively degraded in P3 cardiomyocytes arrested in G1/G0 phase. Stimulation with FGF1 and the p38 inhibitor SB203580 (p38i), which have been shown to efficiently induce P3 rat cardiomyocyte proliferation^28^, increased mAG-hGem(1/110) expression in cardiomyocytes transfected with Ad-mAG-hGem(1/110) by approximately 18-fold compared to the control (Figure 1A and 1B). FGF1/p38i-induced mAG-hGem(1/110) expression could also be easily detected by visual inspection without the need of immunofluorescence analysis. The number of mAG-hGem(1/110)-positive cells per microscopic field was increased by approximately 10-fold compared to the control (Figure 1C and 1D). This suggests that Geminin induction would provide a better live imaging screening system to identify small molecules with the potential to promote cardiomyocyte proliferation than loss of Cdt1.

We therefore chose to use this approach to screen a nuclear receptor ligand library (74 compounds) and an epigenetics screening library (54 compounds) in a 96-well plate format at three different concentrations (Figure 1E) in the presence of 0.2% fetal calf serum (FCS). To this end, we infected P3 rat cardiomyocytes with Ad-mAG-hGem(1/110) (infection efficiency > 90%) using DMSO treatment as negative control. To induce cell cycle activity as a positive control, we treated cells with either 10% FCS, which induced a 5-fold increase in mAG-hGem (1/110)-positive cells per field, or FGF1/p38i, which induced an approximate 10-fold increase in cells expressing this marker. We found that 8 compounds induced at least a 2-fold increase in mAG-hGem-positive cells (Figure 1E and Supplementary information, Table S1). The most potent treatment was 250 nM carbacyclin, which induced an approximate 9-fold increase (Figure 1E-1G). These data suggest that carbacyclin, a known potent agonist of PPARδ, is a previously unknown inducer of mammalian postnatal cardiomyocyte proliferation.

**Figure 2:**
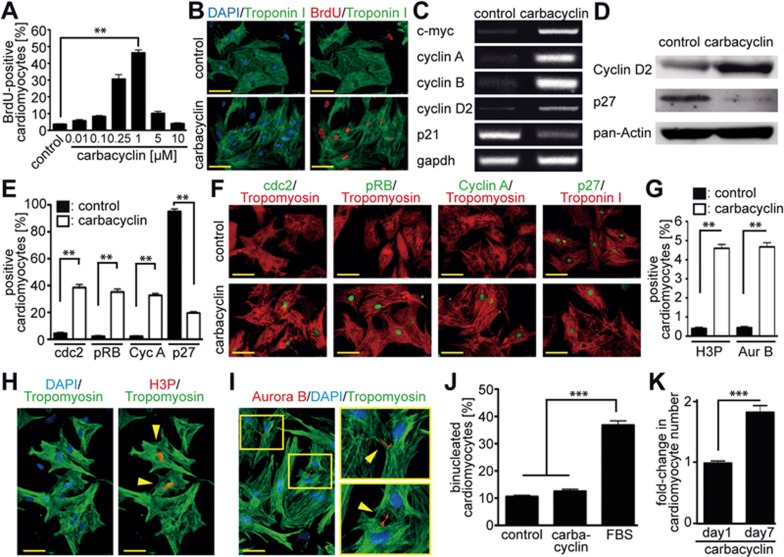
Validation of carbacyclin as an inducer of neonatal cardiomyocyte proliferation. **(A, B)** Representative immunofluorescence images and quantitative analysis (*n* = 6) showing that carbacyclin induces dose-dependent BrdU incorporation (red) into cardiomyocytes (Troponin I, green). **(C-F)** Treatment with carbacyclin induces increased expression of cell cycle promoting factors and decreased expression of cell cycle inhibitors (**C**: RT-PCR; **D**: western blot; **E** and **F**: immunofluorescence analyses at 48 h after stimulation with quantitative analysis, *n* = 6). **(G-I)** Carbacyclin treatment induces mitosis and cytokinesis in neonatal cardiomyocytes. **(G)** Quantitative analysis of H3P- and Aurora B (Aur B)-positive cardiomyocytes after carbacyclin stimulation (1 μM) (*n* = 6). **(H, I)** Representative examples of neonatal cardiomyocytes in mitosis (H3P-positive, red) or cytokinesis (Aurora B-positive, red) (Troponin I, green). DNA was visualized using DAPI (blue). **(J)** Quantitative analysis of binucleation after carbacyclin stimulation after 5 days of treatment (cells were treated on Day 0 and Day 3) (*n* = 4). **(K)** Quantitative analysis of cell count experiments (*n* = 6). ^**^P < 0.01, ^***^P< 0.001. Scale bar = 50 μm.

To determine the optimal concentrations of the 8 identified compounds that would promote progression into S phase, we performed BrdU incorporation assays (Figure 2A, 2B and Supplementary information, Figure S2A-S2G). Carbacyclin was the most potent compound tested and induced BrdU incorporation in a dose-dependent manner with an optimal concentration of 1 μM (46.3% ± 3.8% vs DMSO: 3.6% ± 0.6%, *P* < 0.01, Figure 2A and 2B). Furthermore, mAG-hGem(1/110)-positive P3 cardiomyocytes that had been infected with Ad-mAG-hGem(1/110) and treated with carbacyclin progressed into cytokinesis (Supplementary information, Figure S3A-S3C). Furthermore, carbacyclin induced the expression of positive regulators of cell cycle progression including phospho-RB, cyclin D2, cyclin A, cyclin B, cdc2 and c-myc, and downregulated the cell cycle inhibitors p21 and p27 (Figure 2C-2F). In addition, carbacyclin stimulation increased the number of cardiomyocytes positive for the mitosis/cytokinesis markers phophorylated histone H3 (H3P) and Aurora B by approximately 11-fold within three days (Figure 2G-2I). We observed cardiomyocytes in all stages of the cycle, including the act of division through the breaking of the midbody resulting in two daughter cells. Moreover, the stimulated cells exhibited transient dedifferentiation of the sarcomeric apparatus during mitosis (Supplementary information, Figure S4A). Finally, although carbacyclin treatment did not induce cardiomyocyte binucleation (Figure 2J), it did result in a 2-fold increase in cardiomyocyte cell number within 7 days of culture (Figure 2K). Together, these data demonstrate that carbacyclin induces P3 rat cardiomyocyte proliferation. Notably, carbacyclin had no effect on cell cycle progression of non-myocytes in a non-enriched cardiomyocyte culture (Supplementary information, Figure S4B and S4C).

### Carbacyclin induces cardiomyocyte proliferation *in vitro* via PPARδ

**Figure 3:**
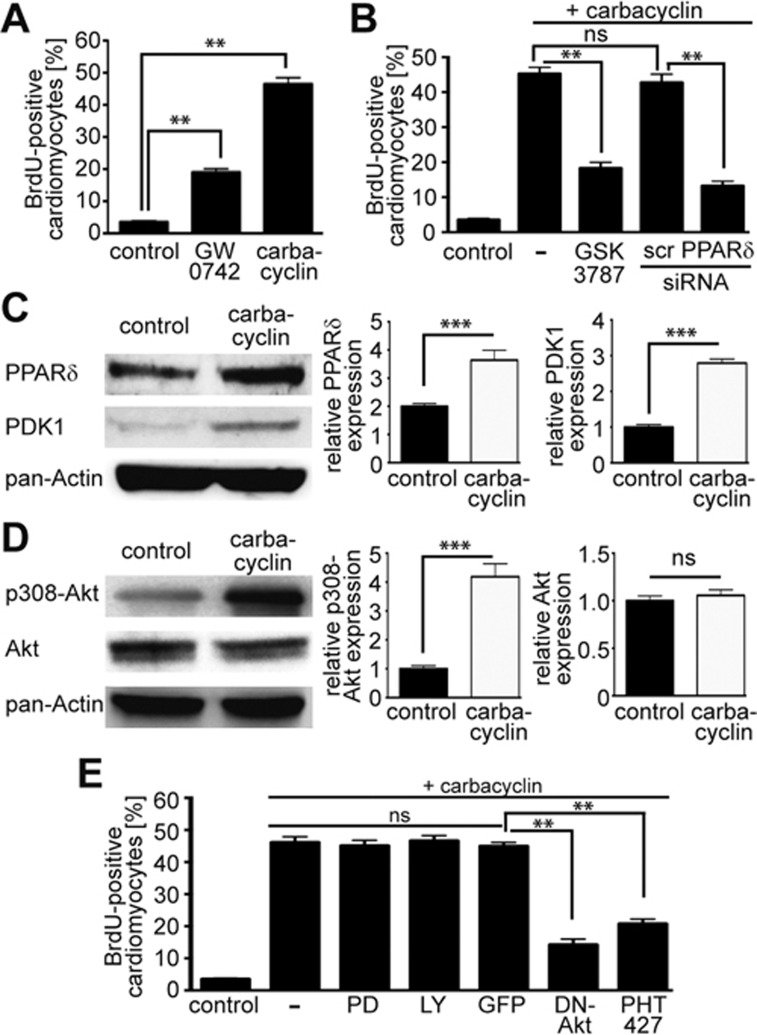
Carbacyclin induces cardiomyocyte proliferation via PPARδ. **(A)** Two agonists of PPARδ, carbacyclin and GW0742 significantly increase DNA synthesis in neonatal cardiomyocytes. **(B)** Inhibition of PPARδ by siRNA or the antagonist GSK3787 markedly reduces the effect of carbacyclin on cardiomyocyte DNA synthesis (scr: scrambled). **(C, D)** Representative examples of western blot analysis including densitometric quantification (*n* = 3) showing that carbacyclin increases the expression of PPARδ at 48 h and PDK1 at 24 h as well as **(C)** phosphorylation of Akt (p308Akt) after 1h **(D)**. **(E)** Akt phosphorylation at position 308 is required for carbacyclin-induced DNA synthesis (dominant negative (DN)-Akt, PHT427) but not PI3 kinase-induced Akt phosphorylation (LY 294002) or ERK activity (PD 98059). ^**^P < 0.01, ^***^P < 0.001.

Carbacyclin is a chemically stable carbocyclic analog of prostacyclin, a known potent agonist of PPARδ. Indeed, treatment with GW0742, another agonist of PPARδ, also substantially increased the number of mAG-hGem(1/110)-positive (Supplementary information, Figure S3D) and BrdU-positive cardiomyocytes (Figure 3A). Moreover, carbacyclin-induced BrdU incorporation (Figure 3B) and mAG-hGem(1/110) expression (Supplementary information, Figure S3E) was markedly reduced by the PPARδ inhibitor GSK3787. In addition, BrdU incorporation could be inhibited by siRNA-mediated knockdown of PPARδ (Figure 3B). These data demonstrate that carbacyclin-mediated cardiomyocyte proliferation requires activation of PPARδ.

It has previously been demonstrated that the PPARδ agonist GW501516 upregulates 3-phosphoinositide-dependent protein kinase-1 (PDK1) expression *in vivo*^29^. Thus, we assessed whether carbacyclin activates PPARδ via PDK1 and phosphorylation of Akt in cardiomyocytes. Carbacyclin stimulation resulted in phosphorylation of Akt at Thr308 as well as upregulation of PPARδ and PDK1 (Figure 3C and 3D). To determine if this pathway is required for carbacyclin-induced cardiomyocyte proliferation, we overexpressed dominant negative (DN)-Akt, which markedly decreased carbacyclin-induced BrdU incorporation (Figure 3E). Moreover, BrdU incorporation was also decreased after addition of 20 μM PHT427, a dual inhibitor of PDK1/Akt. In contrast, neither 20 μM ERK inhibitor PD98059 nor 10 μM PI3 kinase inhibitor LY294002 affected carbacyclin-mediated BrdU incorporation (Figure 3E). In addition, carbacyclin increased BrdU incorporation in human-induced pluripotent stem cell (hiPSC)-derived cardiomyocytes (Supplementary information, Figure S5A and S5B). The addition of 20 μM PHT427 markedly reduced carbacyclin-induced BrdU incorporation in hiPSC-derived cardiomyocytes (Supplementary information, Figure S5B). It also reduced mAG-hGem(1/110) expression in Ad-mAG-hGem(1/110)-infected P3 neonatal cardiomyocytes stimulated with carbacyclin (Supplementary information, Figure S3E). Finally, carbacyclin promoted mitosis and cytokinesis in hiPSC-derived cardiomyocytes (Supplementary information, Figure S5C-S5F). Taken together, these data indicate that carbacyclin promotes cardiomyocyte proliferation via a PPARδ/PDK1/p308Akt pathway.

### PPARδ inhibition reduces cardiomyocyte proliferation during zebrafish heart regeneration

**Figure 4:**
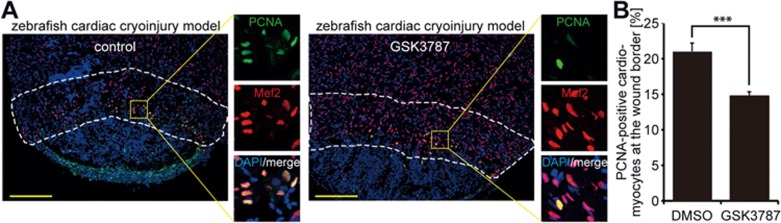
PPARδ activity is required for adult cardiomyocyte cell cycle activity after injury in zebrafish. **(A)** Representative images of cryoinjured zebrafish hearts (7 dpi) treated with DMSO (control) or 5 μM PPARδ inhibitor (GSK3787), stained for PCNA (green) and the cardiomyocyte marker Mef2 (red). Nuclei were visualized using DAPI (blue). Dashed lines highlight the wound border zone used for quantification. Scale bar = 150 μm. **(B)** Quantitative analysis of PCNA-positive cardiomyocytes (DMSO: *n* = 9 hearts; GSK3787: *n* = 10 hearts; ^***^P < 0.001).

Our strategy to find new compounds to induce mammalian heart repair is based on the hypothesis that proliferation signaling pathways are conserved in zebrafish cardiomyocytes, P3 rat cardiomyocytes and a presumptive mononuclear adult mammalian cardiomyocyte subpopulation. If correct, this would infer that PPARδ plays a role in zebrafish heart regeneration. To test this, we subjected zebrafish heart to cryoinjury and 6 days later (6 dpi) inhibited PPARδ activity by a 24 h treatment with GSK3787 before assessing cardiomyocyte proliferation by proliferating cell nuclear antigen (PCNA) expression. The fraction of PCNA-positive cardiomyocytes at the wound border was significantly reduced upon treatment with PPARδ antagonist (Figure 4A and 4B). In addition, inhibition of PPARδ activity led to a reduction in cardiomyocyte mitosis as determined by H3P staining (Supplementary information, Figure S6). We conclude that endogenous PPARδ activity is required for naturally occurring regenerative cardiomyocyte proliferation in zebrafish.

### Activation of PPARδ induces adult cardiomyocyte cell cycle re-entry *in vitro*

**Figure 5:**
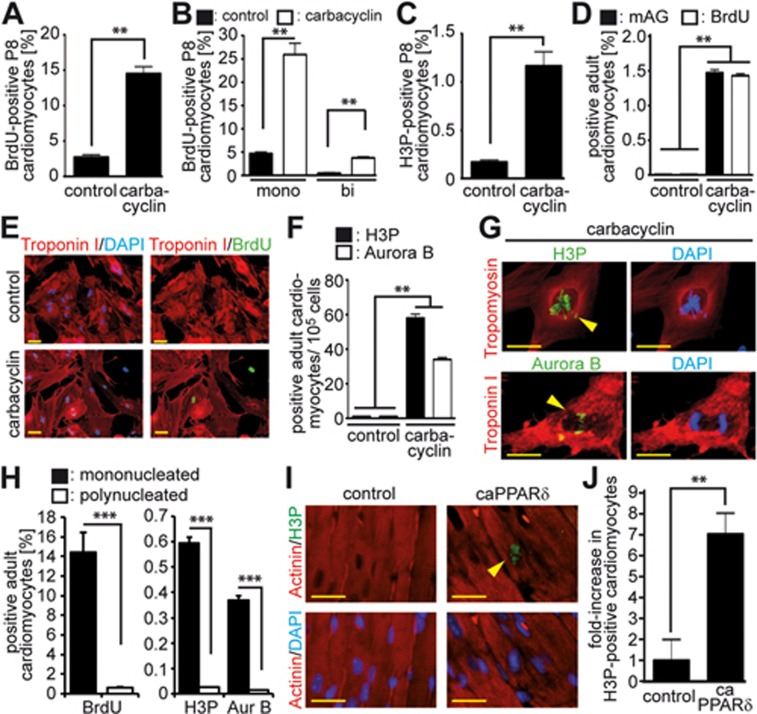
PPARδ activity is sufficient for adult cardiomyocyte cell cycle re-entry. **(A-C)** Carbacyclin induces cell cycle re-entry of P8 cardiomyocytes. Quantitative analysis of BrdU incorporation (**A, B**, *n* = 4) and mitosis based on H3P staining (**C**, *n* = 4) in mono- and binucleated cardiomyocytes. The overall percentage of binucleated cardiomyocytes in these cultures was 41.5% ± 6.1%. **(D, E)** Carbacyclin induces mAG expression after infection with Ad-mAG-hGem(1/110) as well as BrdU incorporation in adult cardiomyocytes. Quantitative analysis (*n* = 6). **(F, G)** Carbacyclin treatment induces mitosis and cytokinesis in adult cardiomyocytes. **(F)** Quantitative analysis (*n* = 6). **(G)** Representative examples of adult cardiomyocytes undergoing mitosis and cytokinesis stained for Tropomyosin or Troponin I (red, cardiomyocyte-specific) and H3P or Aurora B (green). Scale bar = 50 μm. **(H)** Percentage of mono- and binucleated cardiomyocytes within the group of BrdU-positive cardiomyocytes (*n* = 4). **(I)** Representative heart sections from TMCM mice (control) and tamoxifen inducible transgenic mice with cardiomyocyte-restricted overexpression of VP16-PPARδ (caPPARδ) 2 weeks after tamoxifen injection. Sections were stained with anti-Actinin (red, cardiomyocytes-specific) and anti-H3P (green, stains mitotic cells) antibodies. Nuclei were visualized with DAPI. Scale bar = 25 μm. **(J)** Quantitative analysis of **I** (*n* = 4, 3 sections per heart). ^**^P < 0.01, ^***^P < 0.001.

To determine if PPARδ activation can also promote cell cycle progression in more differentiated mammalian cardiomyocytes, we stimulated cardiomyocytes of older animals with carbacyclin. This treatment increased BrdU incorporation in P8 cardiomyocytes *in vitro* by more than 5-fold (14.5% ± 1.7% vs 2.73% ± 0.4%, *P* < 0.01; Figure 5A), with mainly mononucleated cardiomyocytes incorporating BrdU (Figure 5B). The mitotic index (H3P-positive) was increased by almost 7-fold (1.16% ± 0.13% vs 0.17% ± 0.04%, *P* < 0.01; Figure 5C). Carbacyclin treatment of cardiomyocytes from 12-week-old rats increased mAG-hGem(1/110) expression at Day 5 of culture by greater than 150-fold from 0.01% (following DMSO treatment) to 1.5% ± 0.2% (Figure 5D). This resulted in 1.4% ± 0.2% BrdU-positive cardiomyocytes at Day 6 (labeled for the final 5 days of culture) (Figure 5D and 5E). To assess cell division, we analyzed 100 000 adult cardiomyocytes each from 4 independent experiments and found in the control an average of one H3P-positive and (1; 0; 2; 1) and one Aurora B-positive (2; 2; 0; 0) cell. In contrast, after carbacyclin stimulation we detected an average of 58 H3P-positive (64; 55; 60; 54) and 34 Aurora B-positive (31; 34; 39; 32) adult cardiomyocytes (Figure 5F and 5G). Notably, 58.6% ± 5.0% of the BrdU-positive, 58.1% ± 6.4% of the H3P-positive and 61.7% ± 7.3% of the Aurora B-positive adult cardiomyocytes were mononucleated (Figure 5H). Considering that only 5.7% ± 2.2% adult cardiomyocytes were mononucleated in these cultures, carbacyclin induced DNA synthesis in ∼14%, mitosis in ∼0.6% and cytokinesis in ∼0.4% of mononucleated cardiomyocytes compared to control levels of ∼0.6%, ∼0.025% and ∼0.014% in polynucleated adult cardiomyocytes, respectively. Taken together, our data demonstrate that administration of carbacyclin *in vitro* promotes cell cycle progression through S phase, mitosis and cytokinesis in mononuclear adult rat cardiomyocytes and thus at a lower level than in neonatal cardiomyocytes (Figure 2A and 2G), but at a similar level as in P8 cardiomyocytes (Figure 5A-5C).

To determine whether PPARδ activation could induce mitosis in adult cardiomyocytes *in vivo*, we utilized a transgenic mouse model with tamoxifen inducible, cardiomyocyte-restricted overexpression of constitutively active PPARδ (caPPARδ VP16-PPARδ)^27,30^. We refer to tamoxifen inducible αMyHC-Mer-Cre-Mer controls as TMCM mice and animals showing cardiomyocyte-restricted overexpression of VP16-PPARδ as TMVPD mice. Such animals were injected with tamoxifen and 14 days later analyzed for H3P-positive mitotic cardiomyocytes 14 days. Cardiac-specific overexpression of constitutively active PPARδ resulted in a 7-fold higher number of mitotic adult cardiomyocytes (2.33 ± 0.4 vs 0.33 ± 0.3 per section, Figure 5I and 5J). These data suggest that activation of PPARδ promotes cardiomyocyte proliferation *in vitro* and *in vivo*.

### Activation of PPARδ improves heart function after myocardial infarction

**Figure 6:**
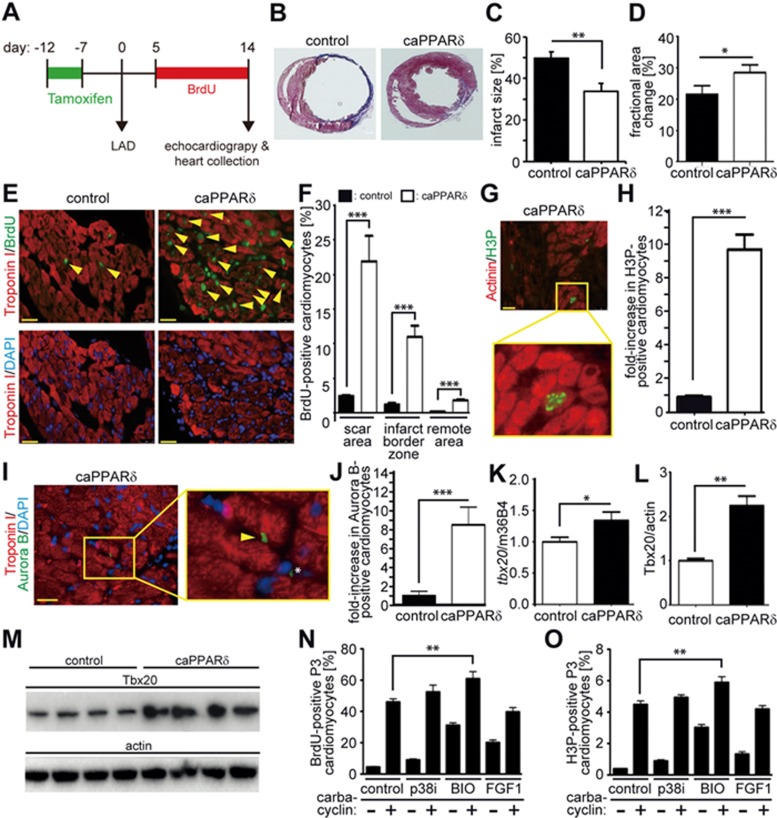
Activation of PPARδ induces cardiomyocyte cell cycle re-entry *in vivo* and rescues cardiac function after myocardial infarction. Cardiomyocyte-restricted overexpression of a constitutively active PPARδ was induced in adult mice utilizing TMVPD mice via tamoxifen injections (caPPARδ). One week after tamoxifen injection, MI was induced via LAD ligation and hearts were analyzed 2 weeks later. **(A)** Experimental design. **(B)** Representative images of hearts from caPPARδ mice and control mice (TMCM) after Masson's Trichrome staining 2 weeks post MI. **(C)** Quantitative analysis of infarct size (*n* = 5) and **(D)** echocardiographic measurement demonstrating improved cardiac morphology and function post MI in caPPARδ mice (*n* = 7) compared to control mice (*n* = 8). **(E)** Representative heart sections showing the scar area from control and caPPARδ mice. Sections were stained with anti-Troponin I (red, cardiomyocytes-specific) and anti-BrdU (green, stains cycling cells) antibodies. Nuclei were visualized with DAPI. Arrowheads: indicate BrdU-positive cardiomyocytes. **(F)** Quantitative analysis of BrdU-positive cardiomyocytes (*n* = 4, 3 sections per heart). **(G)** Representative mitotic cardiomyocyte (Actinin: red, cardiomyocytes-specific; H3P: green, stains mitotic cells) in a heart section from caPPARδ. **(H)** Quantitative analysis of H3P-positive cardiomyocytes (*n* = 4, 3 sections per heart). **(I)** Representative heart section from caPPARδ mice stained for Troponin I (red, cardiomyocytes-specific) and Aurora B (green, stains cells in cytokinesis). Nuclei were visualized with DAPI. Arrow: indicates a dividing cardiomyocyte. Asterix: indicates a dividing non-myocyte. **(J)** Quantitative analysis of Aurora B-positive cardiomyocytes (*n* = 4, 3 sections per heart). **(K)** Quantitative analysis of tbx20 mRNA via real-time PCR. m36B4 was used as control (*n* = 7). **(L, M)** Tbx20 protein expression was assessed via western blot analysis (*n* = 4). ^*^P < 0.05, ^***^P < 0.001. Scale bar = 25 μm. **(N, O)** Quantitative analysis of an additive effect on carbacyclin-induced BrdU incorporation by p38 MAP kinase inhibitor SB203580, fibroblast growth factor 1 (FGF1) or GSK3β inhibitor BIO. ^**^P < 0.01, *n* = 6.

To determine whether PPARδ activation is sufficient to improve the ineffective myocardial repair that takes place after myocardial infarction (MI) in mice, we induced MI by ligation of the left anterior descending artery (LAD) in adult TMCM (control) and TMVPD (caPPARδ) mice (Figure 6A). Analysis of trichrome-stained cross-sections of the heart two weeks post MI showed that the infarct size was reduced in mice overexpressing caPPARδ (Figure 6B and 6C). Echocardiography also revealed that cardiomyocyte-restricted overexpression of caPPARδ in adult TMVPD mice also improved heart function two weeks after MI. caPPARδ mice showed shorter systolic endocardial and epicardial length compared to control TMCM mice, implicating less dilated systolic hearts in caPPARδ expression compared to control animals (Supplementary information, Table S2). Consistently, the change in endocardial area measured in hearts expressing constitutively active PPARδ was greater than that in control hearts (Supplementary information, Table S2). In addition, hearts with constitutively active PPARδ showed an improved percentile fractional area change (FAC%) compared to control hearts, indicating improved cardiac contraction (Figure 6D). Consequently, hearts expressing caPPARδ showed increased endocardial stroke volume and cardiac output (Supplementary information, Table S2).

TMVPD animals with cardiac-specific overexpression of constitutively active PPARδ showed a substantial increase in several parameters 2 weeks after MI relative to control TMCM mice. These included an increase in BrdU incorporation over 10 days in cardiomyocytes in the scar area (> 9-fold), the border zone (> 8-fold) and in the remote zone (11-fold) compared to post-MI TMCM control mice (Scar area: 21.9% ± 3.7% vs 2.41% ± 0.2%, infarct border zone: 10.92% ± 1.7% vs 1.23% ± 0.2%, remote area: 1.76% ± 0.2% vs 0.16% ± 0.03%, Figure 6E and 6F). Moreover, post-MI caPPARδ mice showed a greater than 10-fold increase in mitotic (H3P-positive, 20.3 ± 2 vs 2 ± 0.4 per section) adult cardiomyocytes and a greater than 9-fold increase in cytokinetic (Aurora B-positive, 9 ± 1.15 vs 1 ± 0.29 per section) adult cardiomyocytes compared to post-MI control mice (Figure 6G-6J). Together, these data indicate that overexpression of PPARδ following MI results in a marked beneficial effect by reducing infarct size, stimulating cardiomyocyte proliferation and improving cardiac function. This is further supported by the observation that Tbx20, whose overexpression has recently been demonstrated to promote adult cardiomyocyte proliferation and to improve cardiac function post MI^8^, is significantly upregulated in caPPARδ mice post MI (Figure 6K-6M). In contrast, no change in the number of c-Kit-positive cells was observed suggesting that the recruitment of endogenous heart progenitors plays either no role or a limited role during cardiac repair upon activation of PPARδ (Supplementary information, Figure S7).

To determine whether PPARδ regulates Tbx20 expression at the transcriptional level, we generated a series of pGL3-Basic plasmid constructs for luciferase reporter assays, which contained Tbx20 promoter fragments with or without putative peroxisome proliferators response elements (PPRE) sites (identified by MatInspector software). When PPARδ was co-transfected the reporter activity was significantly increased in constructs containing the −1 150 bp region of the Tbx20 promoter with the PPRE element, suggesting that PPARδ enhances the promoter activity of Tbx20. Co-transfection of PPARγ had no effect (Supplementary information, Figure S8).

Notably, the number of mitotic (H3P-positive) cardiomyocytes 2 weeks post MI in control mice was more than six-fold (2.0 ± 0.4 per section) higher than in uninjured control hearts (0.33 ± 0.3 per section) and more than eight-fold (20.3 ± 2 per section) higher in caPPARδ mice than in uninjured caPPARδ mice (2.33 ± 0.4 per section) (Figures 5 and 6). These data indicate that the MI itself induces a signaling pathway, which promotes the effect of caPPARδ. Previously, it has, e.g., been shown that cardiac injury causes activation of Wnt/β-catenin signaling^31^ that in turn induces cardiomyocyte proliferation *in vitro*^32^ and improves heart function *in vivo*^33^. Notably, it is also known that PPARδ regulates glycogen synthase kinase 3 beta (GSK3β)/β-catenin signaling^34,35^. We therefore assessed whether carbacyclin activates the GSK3β/β-catenin-pathway in cardiomyocytes *in vitro*. We found that carbacyclin stimulation resulted in phosphorylation of GSK3β at Ser9 (Supplementary information, Figure S9A), an increase in nuclear PPARδ and β-catenin (Supplementary information, Figure S9B and S9C), and increased TCF reporter activity (Supplementary information, Figure S9D). Furthermore, overexpression of DN β-catenin (β-cat) or DN-TCF, and addition of 15 μM FH535, an inhibitor of nuclear PPARδ/β-catenin complexes, substantially reduced carbacyclin-induced BrdU incorporation (Supplementary information, Figure S9E). Taken together, these data indicate that PPARδ promotes cardiomyocyte proliferation via GSK3β/β-catenin signaling and that induction of Wnt/β-catenin signaling in the injured heart promotes cardiomyocyte cell cycle entry in caPPARδ mice after MI. This hypothesis is supported by the fact that the addition of 5 μM BIO, a GSK3 inhibitor, but not FGF1 or p38i, markedly increased the number of carbacyclin-induced BrdU-positive and H3P-positive cardiomyocytes in culture (Figure 6N and 6O).

### PPARδ activation post MI has long-term beneficial effects

**Figure 7:**
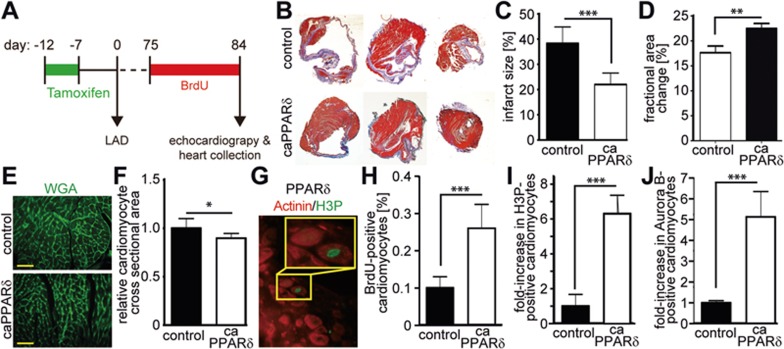
Activation of PPARδ induces cardiomyocyte cell cycle re-entry *in vivo* and rescues cardiac function after myocardial infarction (MI). Cardiomyocyte-restricted overexpression of a constitutively active PPARδ was induced in adult mice utilizing TMVPD mice via tamoxifen injections (caPPARδ). One week after tamoxifen injection, LAD ligation was performed and hearts were analyzed 84 days later. **(A)** Experimental design. **(B)** Representative images of hearts from caPPARδ mice and control mice (TMCM) (three different levels of the same heart) after Masson's Trichrome staining at 84 days post MI. **(C)** Quantitative analysis of infarct size (caPPARδ: *n* = 5, control: *n* = 3) and **(D)** echocardiographic measurement demonstrating improved cardiac function post MI in caPPARδ mice (*n* = 9) compared to control mice (*n* = 8). **(E)** Representative heart sections from control and caPPARδ mice. Sections were stained with wheat germ agglutinin (WGA, green, cell membranes) to determine cardiomyocyte cross sectional area. **(F)** Quantitative analysis of **E** (*n* = 4). **(G)** Representative heart sections from control and caPPARδ mice stained for Actinin (red, cardiomyocytes-specific) and H3P (green, stains mitotic cells). **(H-J)** Quantitative analysis of BrdU-, H3P- and Aurora B-positive cardiomyocytes (*n* = 4, 3 sections per heart). ^*^P < 0.05, ^**^P < 0.01, ^***^P < 0.001. Scale bar = 25 μm.

To determine whether overexpression of caPPARδ following MI has a long-term beneficial effect, we performed a separate double-blinded study inducing MI by LAD ligations in control adult TMCM mice and in mice expressing constitutively active PPARδ (Figure 7A). Analysis of trichrome-stained heart cross sections 84 days post MI showed that the infarct size was reduced by almost 40% in the caPPARδ mice (Figure 7B and 7C). Echocardiography revealed that cardiomyocyte-restricted overexpression of caPPARδ in adult TMVPD mice exerted protective effects on the heart 84 days after MI (Supplementary information, Table S3) resulting in improved FAC% in caPPARδ hearts compared to control hearts (Figure 7D). Of note, as we have shown previously^27^, overexpression of caPPARδ did not result in cardiomyocyte hypertrophy but rather in its inhibition, based on the cardiomyocyte cross sectional area determined by wheat germ agglutinin (WGA) staining (Figure 7E and 7F). Thus, the greater amount of muscle area compared to control hearts (reduced scar area, Figure 7B and 7C) is due to an increased number of cardiomyocytes and not hypertrophy.

At 84 days after MI and upon 10 days of BrdU treatment, the number of BrdU-positive (0.26 ± 0.038 vs. 0.1 ± 0.017, *P* < 0.01), mitotic (H3P-positive) (3 ± 0.29 vs. 0.5 ± 0.15 per section, *P* < 0.01), and cytokinetic (Aurora B-positive) (1.47 ± 0.2 vs. 0.25 ± 0.02 per section, *P* < 0.01) cardiomyocytes was markedly higher in caPPARδ mice compared to control mice (Figure 7G-7J). This indicates active cardiomyocyte proliferation. However, the proliferation indices 84 days after MI was substantially lower than those at 2 weeks post MI suggesting that factors/signaling pathways induced by the MI, such as GSK3β/β-catenin signaling, transiently enhanced the effect of PPARδ activation. Together, these data demonstrate that activation of PPARδ after MI exerts a long-term beneficial effect on heart function.

### Drug-based activation of PPARδ preserves heart function after myocardial infarction

**Figure 8:**
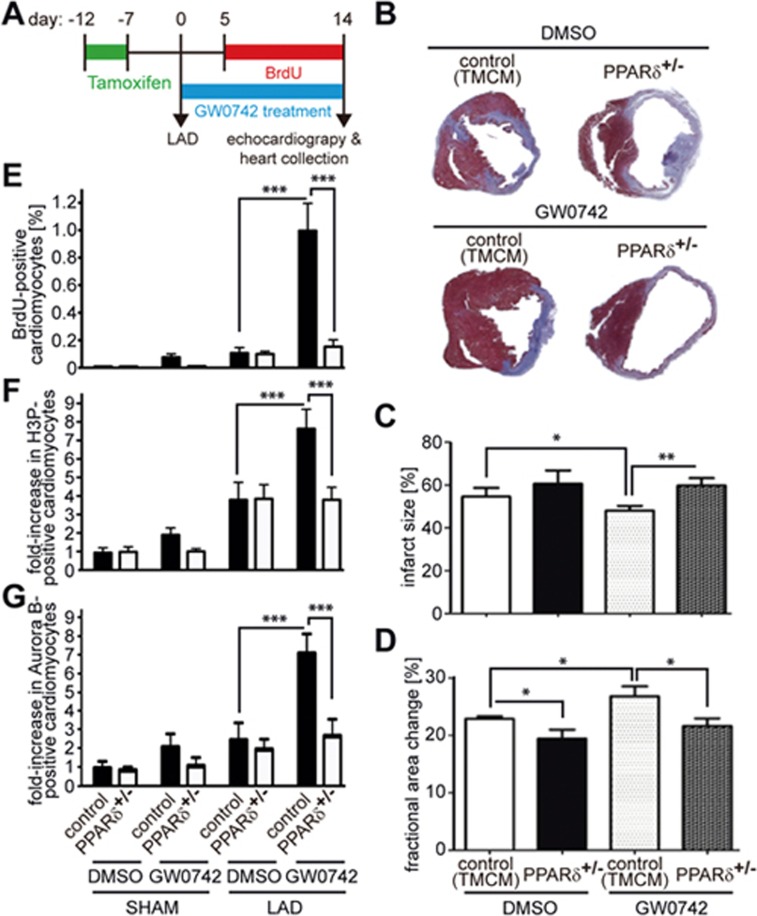
GW0742 treatment induces cardiomyocyte cell cycle progression and rescues heart function and myocardial infarction (MI). **(A)** Experimental design. One week after tamoxifen injections, LAD ligation was performed. Hearts were analyzed after 2 weeks of GW0742 treatment. **(B)** Representative images of PPARδ^+/−^ mice and control mice (TMCM) treated with DMSO or GW0742 after Masson's Trichrome staining 2 weeks post MI. **(C)** Quantitative analysis of infarct size (*n* = 5) and **(D)** echocardiographic measurement demonstrating improved cardiac function post MI in PPARδ^+/−^ mice (DMSO: *n* = 8; GW0742: *n* = 12) compared to control mice (DMSO: *n* = 9; GW0742: *n* = 8). **(E-G)** Quantitative analysis of BrdU-, H3P- and Aurora B-positive cardiomyocytes (*n* = 4, 3 sections per heart). ^*^P < 0.05, ^**^P < 0.01, ^***^P < 0.001.

To determine whether a therapeutic approach toward activating PPARδ induces cardiomyocyte cell cycle progression exerting beneficial effects on the post-MI heart, we performed a third double-blinded study (Figure 8A) utilizing the well-characterized and highly specific PPARδ activator GW0742. Analysis of trichrome-stained heart cross sections 2 weeks post MI showed that the infarct size was substantially reduced in TMCM control mice treated with GW0742 (Figure 8B and 8C). Echocardiography revealed that GW0742 treatment (Supplementary information, Table S4) resulted in improved FAC% in GW0742- compared to DMSO-treated animals 14 days post MI (Figure 8D).

GW0742 treatment markedly increased the number of BrdU-positive (0.93% ± 0.17% vs 0.1% ± 0.04%, *P* < 0.01), mitotic (H3P positive) 4 ± 0.32 vs 2 ± 0.29 per section, *P* < 0.01) and cytokinetic (Aurora B-positive) (2.0 ± 0.17 vs 0.7 ± 0.15 per section, *P* < 0.01) cardiomyocytes compared to DMSO-treated animals 2 weeks after MI (Figure 8E-8G). Notably, improvement in scarring, cardiac function and proliferation markers upon GW0742 treatment was abolished in TMPD^+/^^−^ mice harboring a heterozygous, cardiac-restricted deletion of PPARδ (PPARδ^+/^^−^; Figure 8B-8G, note: homozygous deletion of PPARδ impairs heart function^25^). These data support the notion that PPARδ in cardiomyocytes is required for the effect of PPARδ ligand-induced cardiomyocyte proliferation.

Together, these data suggest that small molecule-based activation of PPARδ after MI induces cardiomyocyte cell cycle progression and exerts a beneficial effect by reducing infarct size and improving cardiac function.

## Discussion

In this study, we modified the Fucci system^12^ by integrating a cardiomyocyte-specific promoter to establish a fluorescence-based live imaging-screening assay to identify new inducers of P3 rat cardiomyocyte proliferation. This system eliminates the need for laborious and expensive techniques such as immunofluorescence staining, incorporation of nucleotide analogs or cell count assays. Most importantly, we identified carbacyclin as a novel potential inducer of P3 cardiomyocyte proliferation. Subsequent proliferation assays have demonstrated that carbacyclin induces proliferation of P3, P8 and preferentially mononuclear adult rat cardiomyocytes via PPARδ. Conversely, inhibition of PPARδ inhibited cardiomyocyte proliferation during zebrafish heart regeneration. Notably, PPARδ activation following MI has both short-term and long-term beneficial effects improving scarring, cardiac function and expression of proliferation markers. Our results confirm the hypothesis that conserved signaling pathways in P3 rat cardiomyocytes are required for cardiomyocyte proliferation during cardiac regeneration in zebrafish and have the potential to promote proliferation of mononuclear adult mammalian cardiomyocyte and cardiac repair.

Our data indicate that PPARδ activation mainly acts on a mononucleated subpopulation of cardiomyocytes that maintains a fetal/neonatal phenotype, as the pro-proliferative effect on cardiomyocytes *in vitro* was markedly decreased with the aging of the stimulated cardiomyocytes. This decline in efficiency with age has previously been reported for several factors resulting in the “subpopulation theory”, which was recently supported by a hypoxia fate-mapping study that identified cycling cardiomyocytes in the adult heart^3,10^.

Previously, it has been shown that PPARδ is expressed in human heart^36^. In addition, activation of PPARδ signaling has been demonstrated to modulate proliferation of other human cell types such as epithelial cells^37,38,39^, endothelial progenitor cells^40^, keratinocytes^15^ and liposarcoma cell lines^41^. In addition, we have shown here that activation of PPARδ signaling promotes proliferation of hiPSC-derived cardiomyocytes. Moreover, PPARδ has the same role in mice and humans^42,43^. These data indicate that activation of PPARδ signaling after MI might be of relevance in the human clinical setting.

While our finding that PPARδ regulates cardiomyocyte proliferation is novel, it is known that PPARδ overexpression has a positive effect on heart function after cardiac injury. Yet there is no report on a positive effect after MI. For example, myocardial injury due to I/R injury, as determined by measuring the infarcted area relative to the area at risk, was significantly reduced by cardiac-specific PPARδ overexpression in adult heart concomitant with increased myocardial glucose utilization^26^. In a subsequent study, Liu and coworkers showed that PPARδ overexpression in adult heart elevates oxidative metabolism concomitant with an increased mitochondrial DNA copy number and an enhanced cardiac performance. While transverse aortic constriction in PPARδ transgenic mice resulted in the same hypertrophic response as in control mice, dilation of the ventricles as well as fibrosis was significantly reduced^27^. Yet, none of these studies has demonstrated that the metabolic changes accounted for the observed beneficial effects. Interestingly, glucose utilization has previously been associated with proliferation and regeneration. For example, proliferative fetal cardiomyocytes, in contrast to postmitotic postnatal cardiomyocytes, utilize mainly glucose^44^ and Lin28a-mediated increase in glycolysis and oxidative phosphorylation enhances tissue repair in several adult tissues^45^. Thus, it will be interesting in future studies to investigate whether alterations of energy metabolism can promote cardiomyocyte proliferation.

That PPARδ activation has a great potential for cardiac repair and is supported by the fact that PPARδ has been associated with wound healing and tissue repair in other organs and tissues^16,17,18,19^. Our observation that PPARδ activation has a stronger effect after MI than in uninjured hearts resulted in the hypothesis that the effect of PPARδ activation is enhanced by GSK3β/β-catenin signaling. Previously, it has been shown that Wnt/β-catenin signaling plays important roles during vertebrate heart development and is re-activated in response to cardiac injury^31,46^. Moreover, inhibition of GSK3β promotes cardiomyocyte proliferation *in vitro*^32^ and is *in vivo* cardioprotective, and induces physiological hypertrophy as well as cardiomyocyte proliferation^33,47^. However, in contrast to PPARδ activation, GSK-3β inhibition leads to excessive fibrosis after MI through hyperactivation of profibrotic TGF-β1-SMAD-3 signaling^33,48,49,50^. This indicates that PPARδ activation and GSK3β inhibition do not share the same downstream signaling pathways. Therefore, it needs to be tested if the pro-regenerative effect of PPARδ activation can be enhanced by GSK3β inhibitors *in vivo*, as we have observed *in vitro*, and whether this treatment is effective if applied after injury or during chronic heart failure. In addition, one has to consider that PPARδ activation has also been associated with the development of tumors in animal studies^24,51^ and proliferative diseases^14,15^. Thus, in order to make use of the PPARδ and the PPARδ/β-catenin synergy in a therapeutic setting, it might be necessary in the future to develop tools for cardiomyocyte-specific activation of PPARδ and β-catenin or for the local delivery of their agonists or modified RNAs at optimized concentrations.

Our data demonstrate that activation of PPARδ signaling is the key for cardiomyocyte proliferation. Even though we initially identified the PPARδ agonist carbacyclin in our screen, we utilized GW0742 to demonstrate the importance of PPARδ signaling in *in vivo* studies, as GW0742 is a well-characterized and highly specific PPARδ activator^52^. In contrast, carbacyclin is a PPARδ activator with prostacyclin effects^53^ that might have obscured the interpretation of the *in vivo* results. However, our *in vitro* data indicate that carbacyclin is more potent than GW0742 in inducing cardiomyocyte proliferation. Carbacyclin might be a more potent PPARδ activator than GW0742, but might also activate additional pro-proliferative pathways. Therefore, future studies are warranted to further determine if non-PPARδ actions of carbacyclin may synergistically optimize its cardiomyocyte proliferation effect *in vivo*.

Previously, we have shown that cardiomyocyte proliferation can be induced by activating β-catenin^32^ or PI3 kinase signaling^28^. Here we show that the PI3 kinase pathway is not involved in cardiomyocyte proliferation induced by activation of PPARδ signaling. This appears surprising as it has been shown that activation of PPARδ stimulates hepatic stellate cell proliferation through the PI3 kinase/protein kinase-C alpha-/beta-mixed lineage kinase-3 pathway^54^. One possible explanation might be that activation of this pathway increases the phosphorylation of p38 MAPK and that inhibition of p38 MAPK abolished activated PPARδ-dependent stimulation of hepatic stellate cell proliferation. In contrast, we have shown that inhibition of p38 MAPK in cardiomyocytes promotes cardiomyocyte proliferation^28^.

Heart diseases, which are often associated with cardiomyocyte loss, represent a significant socio-economic burden. Currently, there is no therapy available to efficiently treat heart disease. Therefore, the development of a cardiomyocyte proliferation screening system and the finding that activation of PPARδ has the potential to improve cardiac function after MI, which could possibly be reinforced with GSK3β inhibitors, is potentially of great therapeutic value.

## Materials and Methods

### Cardiomyocytes isolation, culture and stimulation

The investigation conforms with the Guide for the Care and Use of Laboratory Animals published by the US National Institutes of Health (NIH Publication No. 85-23, revised 1996). The local Committee approved animal experiments for Care and Use of Laboratory Animals (Regierungspräsidium Darmstadt, Gen. Nr. B 2/Anz. 75). Ventricular cardiomyocytes from 3-day-old (P3), 8-day-old (P8) and 12-week-old (adult) Sprague-Dawley rats were isolated and cultured as described^55,56^. Cells were initially cultured for 48-72 h in the presence of 20 μM cytosine-𝒟-arabinofuranoside and 5% horse serum before stimulation to prevent non-myocyte proliferation. Cardiomyocytes were subsequently treated in the presence of serum (0.2% FCS: neonatal, 5% FCS: adult) with DMSO as a negative control, 1 μM Carbacyclin if not stated otherwise (Enzo Life Science) or 100 nM GW0742 (Cayman Chemical). Postnatal cardiomyocytes were stimulated once; adult cardiomyocytes daily while the medium was exchanged every 3 days. As positive control 50 μg/ml FGF1 + 5 μM SB203580 (p38i) were used. Signaling pathway inhibitors were added 1 h before stimulation: 20 μM PHT-427 (Selleckchem), 100 nM GSK3787 (Santa Cruz Biotechnology), 15 μM FH535 (Tocris Bioscience), 10 μM LY294002 (Sigma), 20 μM PD98059 (Cell Signaling).

### Generation and stimulation of hiPSC-derived cardiomyocytes

Human fibroblasts (GM05171, Coriell) were reprogrammed to iPSCs using the Stemgent mRNA Reprogramming Kit (00-0071), the Stemgent microRNA Booster Kit (00-0073) and the Stemgent Stemfect RNA Transfection Kit (00-0069). About 5.0 × 10^4^ fibroblasts were plated onto Matrigel-coated six-well tissue culture plates and transfected with the microRNA cocktail on day 0 of reprogramming. On days 1-3, fibroblasts were transfected with the mRNA reprogramming cocktail daily, which consisted of Oct4, Sox2, Klf4, c-Myc, Lin28 and nGFP mRNAs in a 3:1:1:1:1:1 stoichiometric ratio. Fibroblasts were co-transfected with both the mRNA and microRNA reprogramming cocktails on day 4, and then subsequently only transfected with the mRNA reprogramming cocktail daily from days 5-11. HiPSC colonies were manually selected from the fibroblast cultures after 2-3 days and cultured on Matrigel-coated 12-well plates. hiPSCs were cultured in E8 media and were split every 4 days. Differentiation along the cardiac lineage was induced as previously described^57^. Briefly, hiPSCs were maintained in E8 media and passaged every 4 days onto Matrigel-coated plates. On day 0 (start of differentiation) hiPSCs were treated with 1 mg/ml Collagenase B (Roche) for 1 h, or until cells dissociated from plates, to generate embryoid bodies (EBs). Cells were collected and centrifuged at 1 200 r.p.m. for 3 min, and resuspended as small clusters of 50-100 cells by gentle pipetting in differentiation media (RPMI (Gibco), 2 mM ℒ-glutamine (Invitrogen), 4 × 10^4^ monothioglycerol (MTG, Sigma), 50 μg/ml ascorbic acid (Sigma)). Differentiation media was supplemented with 2 ng/ml BMP4 and 3 μmol Thiazovivin (Milipore) on day 0. EBs were maintained in 6 cm dishes at 37 °C in 5% CO_2_, 5% O_2_ and 90% N_2_. On day 1, media was changed to differentiation media supplemented with 20 ng/ml BMP4, 20 ng/ml Activin A, 5 ng/ml bFGF (all R&D Systems) and 1 μmol Thiazovivin (Milipore). On day 3, EBs were harvested and washed once with DMEM (Gibco). Media was changed to differentiation media supplemented with 5 ng/ml VEGF (R&D Systems) and 5 μmol/l XAV (Stemgent). On day 5, media was changed to differentiation media supplemented with 5 ng/ml VEGF (R&D Systems). After day 8, media was changed to differentiation media without supplements and was renewed every 3-4 days. hiPSC-derived cardiomyocytes were then stimulated at day 23 with carbacyclin.

### Screening

P3 rat cardiomyocytes were seeded in 100 μl medium at a density of 15 000 cells per 96 well for 2 days. Then cells were infected with Ad-mAG-hGem(1/110). After 24 h they were washed and treated with compounds dissolved in DMSO, and 0.2% FCS (Nuclear Receptor Ligand Library, 74 compounds, Enzo Life Science; Epigenetics Screening Library, 54 compounds, Cayman Chemicals). AG expression was analyzed every 12 h (for quantitative analysis a random field of around 100 cells was evaluated) for the following 4 days by visual inspection using a Leica fluorescence microscope. The maximal number of mAG-hGem(1/110)-positive cells was used to normalize the data against the DMSO-treated control as a fold change. Hit compounds were defined as those giving an effect greater than 2-fold.

### Adenovirus infection

mCherry-hCdt1(30/120) and mAG-hGem(1/110) were PCR amplified from lentiviral vectors^12^ and cloned into pAlpha-MyHC (clone 26) using *Sal*I^58^ to generate adenoviruses (Sirion Biotech GmbH). Cardiomyocytes were infected 1 (adult) or 2 (P3) days after isolation at a multiplicity of infection (MOI.) of 400. Infection efficiency of cardiomyocytes was > 90%. For inhibition experiments, cultures were infected with adenoviruses expressing DN-Akt (200 MOI)^59^, DN-β-catenin and DN-TCF4 (both 100 MOI, Vector Biolabs) 2 days after isolation. Cells were washed after 24 h and treated.

### Immunofluorescence staining

Staining was performed as described^28,55,56^. Primary antibodies: mouse monoclonal anti-mCherry (1:200, Clontech, 632543 or 1:50, DSHB, 3A11), anti-Tropomyosin (1:200, Sigma, T9283), anti-sarcomeric alpha Actinin (1:100, Abcam, ab9465), anti-Aurora B (1:200, BD Transduction Laboratories, 611083), anti-p27 (1:50, BD Transduction Laboratories, 610242), anti-Ki67 (1:250, Abcam, ab8191), rabbit polyclonal anti-Troponin I (sc-15368), anti-Cyclin A (sc-751), anti-cdc2 (sc-954), anti-Geminin (sc-13015) (all 1:50, Santa Cruz), anti-phospho-Histone H3 (Ser10) (1:200, Millipore, 06-570), anti-pRb807/811 (1:100, Cell Signaling, 9308), anti-mAG (1:300, MBL, PM011), anti p27 (1:50, SantaCruz, sc-528), anti-survivin (1:50, Novus, NB500-201K8), rat monoclonal anti-BrdU (1:100, Abcam, ab6326) and goat polyclonal anti c-kit (1:100, R&D Systems, AF1356). Immune complexes were detected with ALEXA 488- or ALEXA 594-conjugated secondary antibodies (1:200; Molecular Probes). DNA was visualized with DAPI (4′, 6′-diamidino-2-phenylindole, 0.5 g/ml). For BrdU, cells were cultured in 30 μM BrdU (neonatal: last 24 h, adult: last 5 days).

### RT-PCR analysis

Total RNA was isolated using the RNeasy Kit (Qiagen). RT reaction was performed using the oligo(dT) primer (Qiagen). PCR was performed according to standard protocols. Primers: p21: forward 5′-AGGCAGACCAGCCTAACAGA-3′, reverse, 5′-CAGCACTA AGGAGCCTACCG-3′, c-myc: forward (F) 5′-CGAGCTGAAGCGTAGCTTTT-3′, reverse (R) 5′-CTCGCCGTTTCCTCAGTAAG-3′, β-catenin: F 5′-ACAGCACCTTCAGCACTCT-3′, R 5′-AAGTTCTTGGCTATTACGAC-3′, PPARδ: F 5′-GAACAGCCACAGGAGGAGAC-3′, R 5′-CCCATCACAGCCCATCTG-3′, cyclin B: F 5′-GCGTAAAGTCAGCGAACAGTCAAG-3′, R 5′-gcGGAGAGGGAGTATCAACCAAA-3′, cyclin A: F 5′-GCGTATTTGCCATCGCTTATTGCT-3′, R 5′-GCGCTGTGGTGCTTTGAGGTAGGT-3′, cyclin D2: F 5′-AAGAGAGAGGCGTGTTCGTC-3′, R 5′-TTCCTTCTTGGGTTCAATGC-3′, gapdh: F 5′-CAGAAGACTGTGGATGGCCC-3′, R 5′-AGTGTAGCCCAGGATGCCCT-3′.

### Western blotting

Protein extracts were prepared and western blot analyses performed as described^60^. Briefly, equal amount of proteins was resolved by 10% Novex Bis-Tris Gels (Invitrogen) and blotted onto nitrocellulose membranes. Membranes were blocked (5% non-fat dry milk (DM) or 5% BSA in Tris-buffered saline (TBS: 10 mM Tris-HCl (pH 7.5), 150 mM NaCl) with 0.1% Tween, 1 h, RT) and incubated with primary antibodies diluted in 5% milk/TBS/T and/or 5%BSA/TBS/T (overnight, 4 °C): rabbit polyclonal anti-Cyclin D2 (1:1 000, sc-593), anti-PPARδ (1:500, sc-7197) (both Santa Cruz, DM), anti-phospho-GSK-3 (DM, 9336), anti-pan-actin (DM, 4968), anti-PDK1 (BSA, 3062) (all 1:1 000, Cell Signaling), rabbit monoclonal anti-β-catenin (1:1 000, BSA, 9582), anti-GSK-3β (1:1 000, DM, 9315), mouse monoclonal phospho (308) anti-Akt (1:1 000, BSA, 9275), anti-Akt (1:1 000, DM, 2920) (all Cell Signaling), anti-KIP/p27 (1:2 500, DM, 610241), anti-PARP (1:1 000, DM, 611038) (all BD Transduction Laboratories). Antigen/antibody complexes were visualized using horseradish peroxidase-conjugated secondary antibodies (Amersham) and Super Signal@ ECL detection system (Bio-Rad).

### RNA interference

For siRNA knockdown, cardiomyocytes were transfected 48 to 72 h after seeding by lipofectamine RNAiMAX kit (Invitrogen) with validated siRNAs or All Stars Negative Control siRNA (Qiagen) (100 nM), washed after 4 h, and stimulated after 48 h. The efficiency of siRNAs was verified by RT-PCR.

### Luciferase assay

TOPflash or FOPflash reporter plasmids (2 μg, Upstate Signaling) were co-transfected with 2 μg pGL4.75 (hRluc/CMV) vector (Promega) into 2 Mio cells (Nucleofactor Kit, Amaxa, efficiency of transfection: 30%^61^). Cells were cultured 3 days in 24-well plates and subsequently stimulated with compounds for 24 h. Reporter activity was measured by using the Dual Luciferase Assay System (Promega). TOPflash or FOPflash activity was normalized to Renilla luciferase activity of pGL4.75, an internal standard for transfection efficiency.

### Tbx20 promoter analysis

A series of Tbx20 promoter reporter constructs and plasmids expressing either PPARδ or PPARγ were co-transfected into HEK293 cells using Lipofectamine 2000 (Invitrogen). Forty-eight hours after transfection, the cells were lysed with positive lysis buffer (Promega) and the firefly and renilla luciferase activities in the lysate were determined using a luminometer. The Tbx20 promoter luciferase activities were normalized to renilla luciferase activities.

### Zebrafish cryoinjury and PPARδ inhibitor (GSK3787) treatment

All procedures involving zebrafish were approved by local animal experiments committees and performed in compliance with animal welfare laws, guidelines and policies, according to national and European law. Wild-type Gol or *myl7*:GFP^twu34Tg^ zebrafish^62^ aged ∼8 months were used. Heart cryoinjury was performed as described previously^63^ except that a liquid nitrogen cooled copper filament was used instead of dry ice to induce cryoinjury. After the injury, fish were maintained under standard housing conditions till 6 days post injury (dpi). They were then kept in aquarium water containing either DMSO solvent or PPARδ inhibitor (GSK3787; 5 μM) for a duration of 24 h. At 7 dpi, hearts were harvested, fixed and cryosectioned as described previously^63^.

### Zebrafish immunofluorescence and cell quantification

Primary antibodies used were anti-PCNA (1:2 000; Dako #M0879), anti-mef2 (1:50; Santa Cruz Biotechnology #sc-313) and anti-H3P (1:1 000, Cell Signaling Technology #9706). DAPI was used to stain nuclei. Images shown are single optical planes acquired using a Leica SP5 confocal microscope. For quantification of PCNA positive cardiomyocytes, the percentage of Mef2-positive cells also positive for PCNA was quantified within a zone that extended 150 μm from the wound border. For each heart 2-3 sections displaying the biggest wounds were analyzed. H3P-positive cardiomyocytes were quantified on all heart sections, which contained an injury area (average eight sections per heart). In this case the total cardiomyocyte number in the border zone was estimated by determining the average density of cardiomyocytes per μm^2^ in three separate areas (size: 75 μm^2^) and by multiplying this number by the total border area size.

### PPARδ mouse model, myocardial infarction, echocardiography and BrdU treatment

All experimental procedures were conducted in accordance with the Guide for Care and Use of Laboratory Animals, and were approved by the Institutional Animal Care and Use Committee of the University of Alabama at Birmingham (UAB). The utilized transgenic mouse line TMVPD line allows tamoxifen inducible cardiomyocyte-restricted overexpression of a constitutively active mutant *PPARδ* gene (VP16-PPARδ) and has been described previously^27,30,64^. This line was generated by crossing a transgenic line expressing VP16-PPARδ under the control of the human cytomegalovirus immediate early enhance/chicken β-actin promoter with tamoxifen inducible αMyHC-Mer-Cre-Mer (TMCM) transgenic mice. TMPD^+/^^−^ mice harbor a heterozygous, cardiac-restricted deletion of PPARδ, which were generated by crossing floxed PPARδ mice with TMCM mice. Tamoxifen injection once daily for 5 days induced cardiomyocyte-restricted PPARδ overexpression (1.8-fold^27^). MI was induced 1 week after tamoxifen administration by ligation of the left anterior descending coronary artery (LAD). Coronary artery ligation was performed on 3 months old, male mice. Briefly, mice were anesthetized with isoflurane and a catheter was inserted into the trachea. The chest was opened through a left parasternal incision to expose the heart at the left 3rd-4th intercostal space. The pericardium was opened, and ligations were made on the left anterior descending coronary artery using 8-0 silk sutures. The lungs were slightly overinflated to assist in removal of air in the pleural cavity. For the drug-based approach, GW0742 treatment started on the same day as the surgery by subcutaneous injection at a dose of 1 mg/kg until sacrifice, using 10% DMSO/Saline as vehicle. The *in vivo* effect of GW0742 on activating PPARδ target genes has previously been reported^65^. For BrdU (Sigma) labeling, BrdU was dissolved in drinking water at a final concentration of 1 mg/ml and provided to the mice daily for 10 days before harvesting tissue for paraffin sections. BrdU-containing water bottles were shielded from light to prevent BrdU degradation and the water was replaced twice per week. A high-resolution echocardiograph system (VisualSonic VEVO 770 System) was used to assess cardiac structure/function *in vivo* with a 35 MHz probe before terminal experiments. All data and images were saved and analyzed by an Advanced Cardiovascular Package Software (VisualSonic VEVO 770 System) under EKV mode.

### Immunostaining and histology of heart sections

Mice were anesthetized and hearts were perfused (cardioplegic buffer), fixed (4% paraformaldehyde), embedded (paraffin) and sectioned (5 μm thick). Following antigen retrieval in boiling EDTA Buffer (1 M EDTA, pH 8.0, 8 min), sections were cooled to room temperature (RT), blocked (5% goat serum/0.2% Tween-20/PBS, 30 min), incubated overnight at 4°C with rabbit anti-phospho-Histone H3 (Ser10) (1:200, Millipore, 06-570) and mouse anti-sarcomeric alpha Actinin (1:100, Abcam, ab9465) or mouse anti-Aurora B (1:200, BD Transduction Laboratories, #611083) and rabbit anti-Troponin I (1:50, Santa Cruz, sc-15368) washed with PBS, and incubated with corresponding secondary antibodies conjugated to Alexa Fluor 488 and Alexa Fluor 594 (Invitrogen). Nuclei were visualized with DAPI. Hematoxylin/eosin and Masson's Trichrome staining were used to assess cardiac morphology and fibrosis. Infarct size was calculated according to the formula: (length of coronal infarct perimeter (epicardial + endocardial)/total left ventricle coronal perimeter (epicardial + endocardial)) × 100^66^. For the serial section analysis in Figure 5, the software MIQaunt was utilized^67^.

### Wheat germ agglutinin staining

To determine cardiomyocyte cross sectional areas, slides were rinsed three times after deparaffinization in PBS and then incubated for 1 h at RT with WGA conjugated to Alexa Fluor 488 (50 μg/ml, Invitrogen, CA, USA). Slides were then rinsed in PBS and mounted in Vectashield (Vector Labs, CA, USA). To quantify the size of cells, images at 20× magnification were analyzed using ImageJ. Quantitative analyses involved counting of multiple fields from four independent hearts per group, and three sections per heart (∼50 cells per field assessed, total ∼250 cells per sample).

### Statistical analysis

For immunofluorescence analyses, 50 cardiomyocytes in five random fields of two different subpopulations were counted per experiment (total: 500 cardiomyocytes) based on previous experiences. Data of at least three independent experiments are expressed as mean ± SEM. For animal studies, group sizes (at least six) were estimated according to previous experience and power calculation. Before the experiment and data analysis, animals were randomized to new cages by a scientist who was not involved in this project. Thus, the investigator who performed surgery and echocardiographic assessment was not able to recognize group information during the experiment and data analysis. Animals were excluded from the study based on pre-established criteria: (1) genotyping errors; (2) transgenic induction errors and (3) surgical errors. Results were analyzed by Graph Pad Prism (version 4.00, Graph Pad Software). During data analysis, the investigator was blinded to the group allocation. All group information was revealed after data analysis was finalized. Statistical significance was determined using a two-tailed Student's *t*-test and analysis of variance. The values of *p* < 0.05 were considered statistically significant.

## Author Contributions

AM performed, assisted by HVR, all *in vitro* experiments and all histological analyses except the analysis of scar area. AM, YD, LH, TK and KY performed all *in vivo* experiments and the analysis regarding scaring and Tbx20 expression. QL performed the Tbx20 promoter analysis. MDV performed all zebrafish experiments. NW and ND generated hiPSC-derived cardiomyocytes. FBE, GW, QY and AM designed the experiments and wrote the manuscript. All authors were involved in the analysis and interpretation of the data.

## Competing Financial Interests

The authors declare no competing financial interests.

## Supplementary Information

Supplementary information, Figure S1 Screening strategy.

Supplementary information, Figure S2 Validation of positive hits as inducer of cardiomyocyte cell cycle re-entry.

Supplementary information, Figure S3 Validation of the adenovirus-based Fucci system in P3 rat cardiomyocytes.

Supplementary information, Figure S4 Indicators of cardiomyocyte cell division.

Supplementary information, Figure S5 Carbacyclin induces proliferation of hiPSC-derived cardiomyocytes via PDK1.

Supplementary information, Figure S6 PPARδ activity is required for adult cardiomyocyte proliferation in regenerating zebrafish hearts.

Supplementary information, Figure S7 Activation of caPPARδ does not induce an increase in c-kit^+^ cells *in vivo* after MI.

Supplementary information, Figure S8 PPARδ regulates Tbx20 expression at the transcriptional level.

Supplementary information, Figure S9 Carbacyclin activates GSK3β/β-catenin signaling.

Supplementary information, Table S1 Magadum *et al*. Table S1

Supplementary information, Table S2 Magadum *et al*. Table S2

Supplementary information, Table S3 Magadum *et al*. Table S3

Supplementary information, Table S4 Magadum *et al*. Table S4
