# Supplementary material for: Live cell screening platform identifies PPARδ as a regulator of cardiomyocyte proliferation and cardiac repair
Source: Cell Res. 2017 Jun 16;27(8):1002–19. doi: 10.1038/cr.2017.84 (PMC5539351; doi:10.1038/cr.2017.84)
Supplement: Supplementary information, Figure S1 — Screening strategy. [file cr201784x1.pdf]

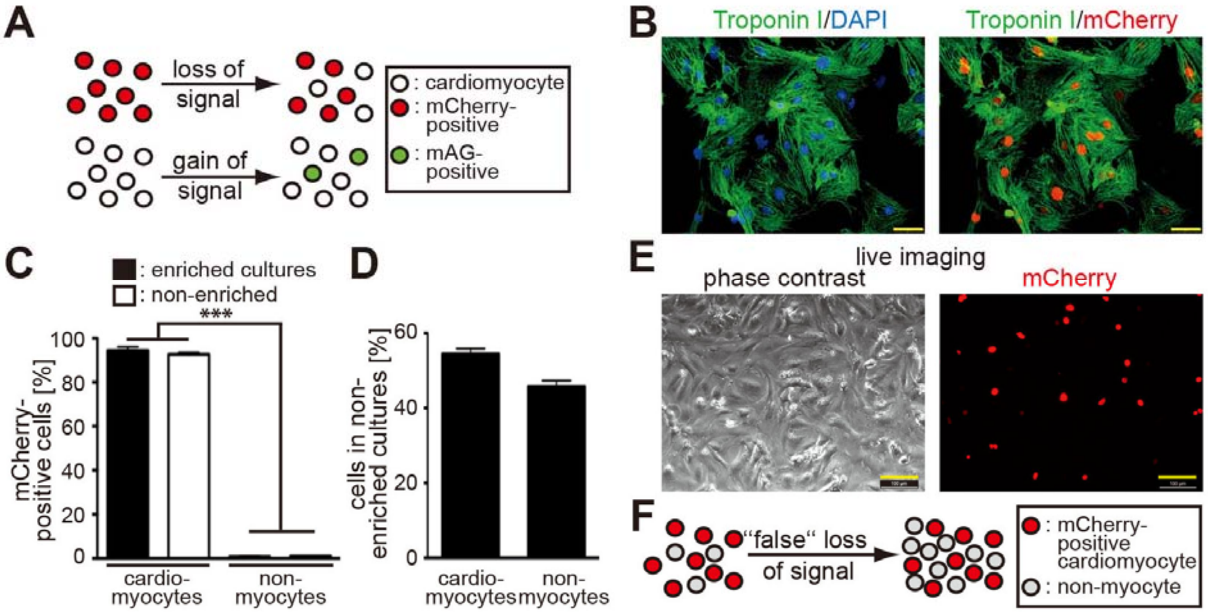

Supplementary information, Figure S1 Screening strategy. **(A)** Schematic representation of possible screening approaches in which induction of proliferation is associated with a loss or gain of signal. **(B)** Representative example for enriched (1 h preplating) P3 rat cardiomyocyte cultures infected with Ad-mCherry-hCdt1(30/120) stained for mCherry expression (red) and counterstained against cardiomyocyte-specific Troponin I (green). DAPI was used to visualize nuclei (blue). **(C)** Quantitative analysis of mCherry-positive cells in enriched and non-enriched (no preplating) cardiomyocyte cultures (n = 6). **(D)** Quantitative analysis of cardiomyocytes and non-myocytes in non-enriched cultures (n = 4). **(E)** Representative live pictures of a control neonatal cardiomyocyte culture infected with Ad-mCherry-hCdt1(30/120) (red). **(F)** Schematic representation of the effect of drugs inducing non-myocyte proliferation mocking loss of signal.
