# Supplementary material for: Live cell screening platform identifies PPARδ as a regulator of cardiomyocyte proliferation and cardiac repair
Source: Cell Res. 2017 Jun 16;27(8):1002–19. doi: 10.1038/cr.2017.84 (PMC5539351; doi:10.1038/cr.2017.84)
Supplement: Supplementary information, Figure S2 — Validation of positive hits as inducer of cardiomyocyte cell cycle re-entry. [file cr201784x2.pdf]

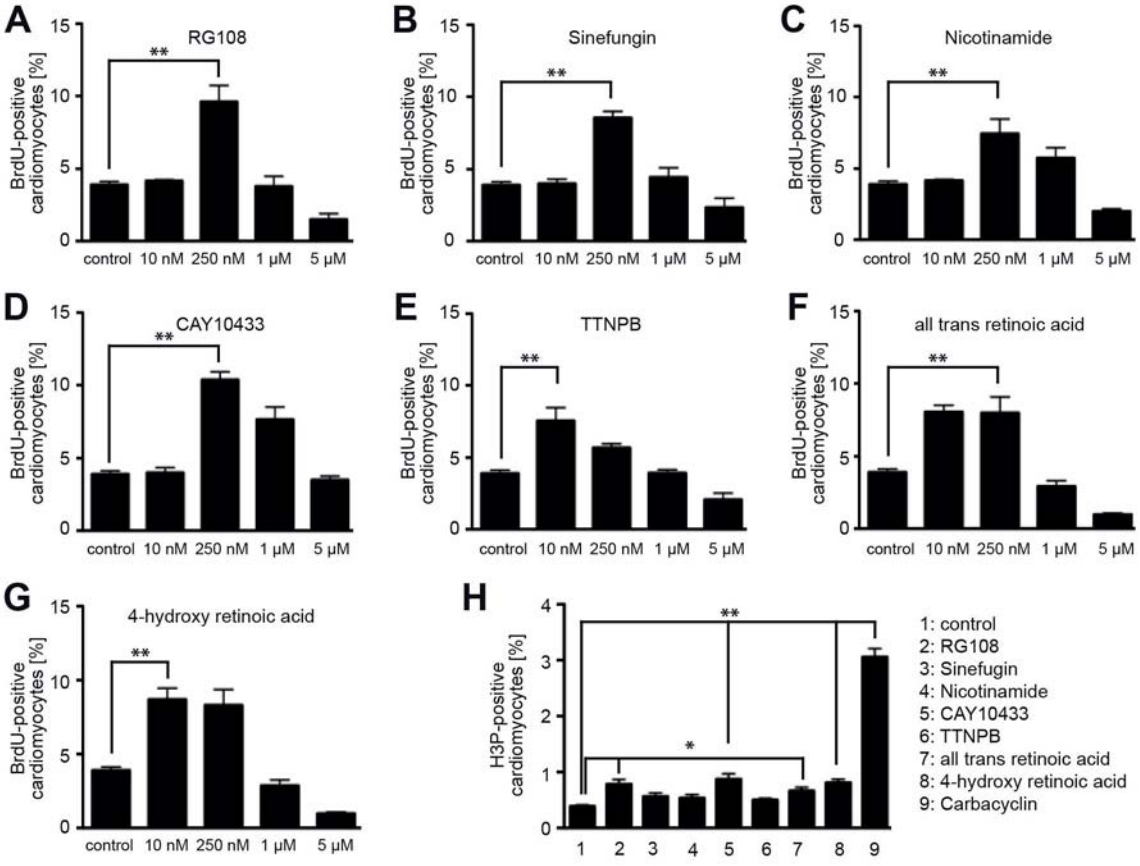

Supplementary information, Figure S2 Validation of positive hits as inducer of cardiomyocyte cell cycle re-entry. **(A-G)** Quantitative analysis of dose-dependent incorporation of BrdU in neonatal cardiomyocytes (*n* = 4). **(H)** Quantitative analysis of H3P-positive cardiomyocytes (*n* = 4). For each compound the most efficient concentration regarding BrdU incorporation was used (for 7 and 8: 10 nM). *$P < 0.05$; **$P < 0.01$.
