# Supplementary material for: Live cell screening platform identifies PPARδ as a regulator of cardiomyocyte proliferation and cardiac repair
Source: Cell Res. 2017 Jun 16;27(8):1002–19. doi: 10.1038/cr.2017.84 (PMC5539351; doi:10.1038/cr.2017.84)
Supplement: Supplementary information, Figure S3 — Validation of the adenovirus-based Fucci system in P3 rat cardiomyocytes. [file cr201784x3.pdf]

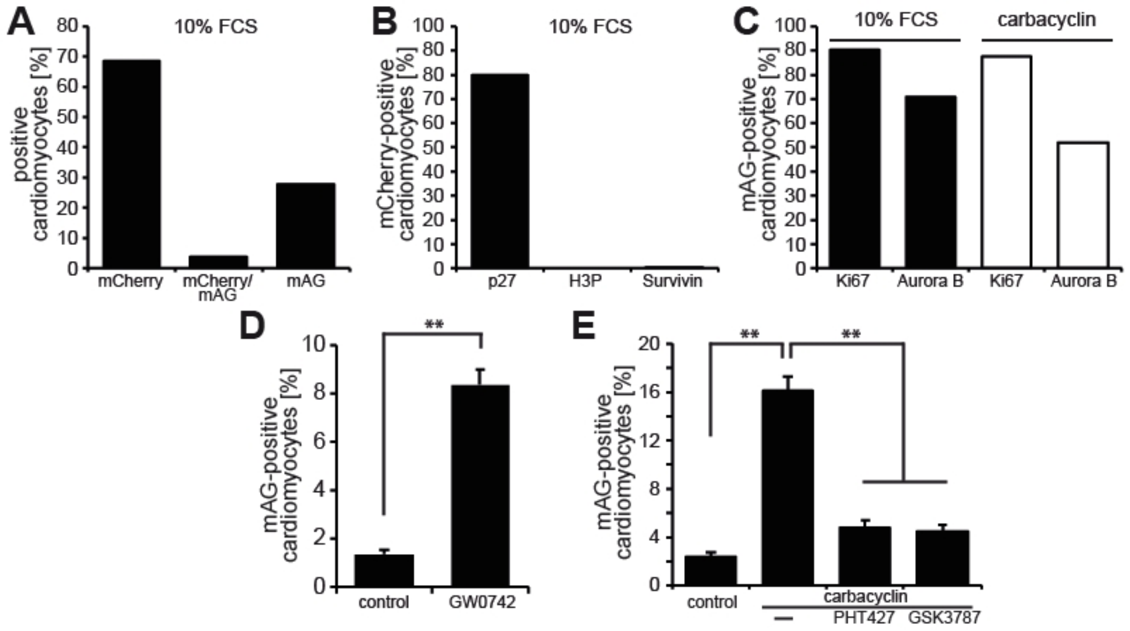

Supplementary information, Figure S3 Validation of the adenovirus-based Fucci system in P3 rat cardiomyocytes. **(A)** Cardiomyocytes co-infected with Ad-mCherry-hCdt1(30/120) and Ad-mAG-hGem(1/110) were stimulated for 48 hours with 10% FCS and subsequently stained for mCherry and mAG and quantified (> 2000 cells). As expected only a few cells, supposedly in G1/S, co-express mCherry and mAG. **(B)** Cardiomyocytes co-infected with Ad-mCherry-hCdt1(30/120) were stimulated for 48 hours with 10% FCS and subsequently stained for mCherry and p27, a marker for G0/G1 phase, or H3P and survivin, marker for mitosis and cytokinesis. The data confirm that Cdt1 is only stable in G0/G1 phase (80 to 285 cells). **(C)** Cardiomyocytes co-infected with Ad-mAG-hGem(1/110) were stimulated for 48 hours with 10% FCS or 1 µM carbacyclin and subsequently stained for mAG and Ki67 or survivin, marker for proliferation and cytokinesis. The data confirm that Geminin is stable in S to G2/M phase (146 to 200 cells). **(D)** Quantitative analysis of mAG expression in P3 cardiomyocytes (*n* = 3) infected with Ad-mAG-hGem(1/110) (green) after stimulation with the PPARδ agonist GW0742 (*n* = 3). **(E)** Quantitative analysis of mAG expression in Ad-mAG-hGem(1/110) infected P3 cardiomyocytes after stimulation with carbacyclin in the absence and presence of the dual inhibitor of PDK1/Akt or the PPARδ inhibitor GSK3787 (*n* = 3). ***P* < 0.01.
