# Supplementary material for: Live cell screening platform identifies PPARδ as a regulator of cardiomyocyte proliferation and cardiac repair
Source: Cell Res. 2017 Jun 16;27(8):1002–19. doi: 10.1038/cr.2017.84 (PMC5539351; doi:10.1038/cr.2017.84)
Supplement: Supplementary information, Figure S4 — Indicators of cardiomyocyte cell division. [file cr201784x4.pdf]

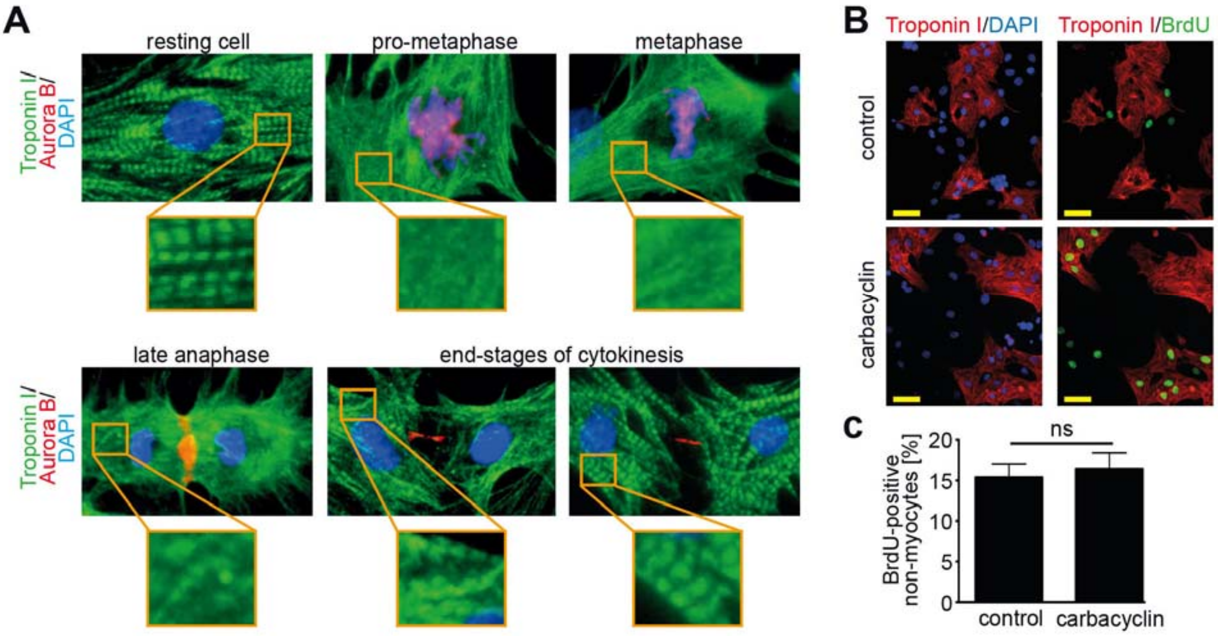

Supplementary information, Figure S4 Indicators of cardiomyocyte cell division. **(A)** Disassembly of myofibrils during carbacyclin-induced cardiomyocyte cell division. The extent of sarcomere disassembly was assessed in carbacyclin-treated cardiomyocytes using Troponin I (green). Cells were stained for Aurora B (red) and DNA (DAPI, blue) to assess the cell cycle status. Striations were apparent in resting/unstimulated cells. During pro-metaphase, sarcomeres became disassembled and were reassembled during late anaphase. **(B)** Representative examples of non-enriched cardiomyocyte cultures stimulated with DMSO or carbacyclin stained for BrdU (green) and Troponin I (red, cardiomyocyte-specific). DNA was visualized using DAPI (blue). Scale bars: 50 µm. **(C)** Quantitative analysis of BrdU-positive non-myocytes in **B**. ns: not significant (*n* = 4).
