# Supplementary material for: Live cell screening platform identifies PPARδ as a regulator of cardiomyocyte proliferation and cardiac repair
Source: Cell Res. 2017 Jun 16;27(8):1002–19. doi: 10.1038/cr.2017.84 (PMC5539351; doi:10.1038/cr.2017.84)
Supplement: Supplementary information, Figure S5 — Carbacyclin induces proliferation of hiPSC-derived cardiomyocytes via PDK1. [file cr201784x5.pdf]

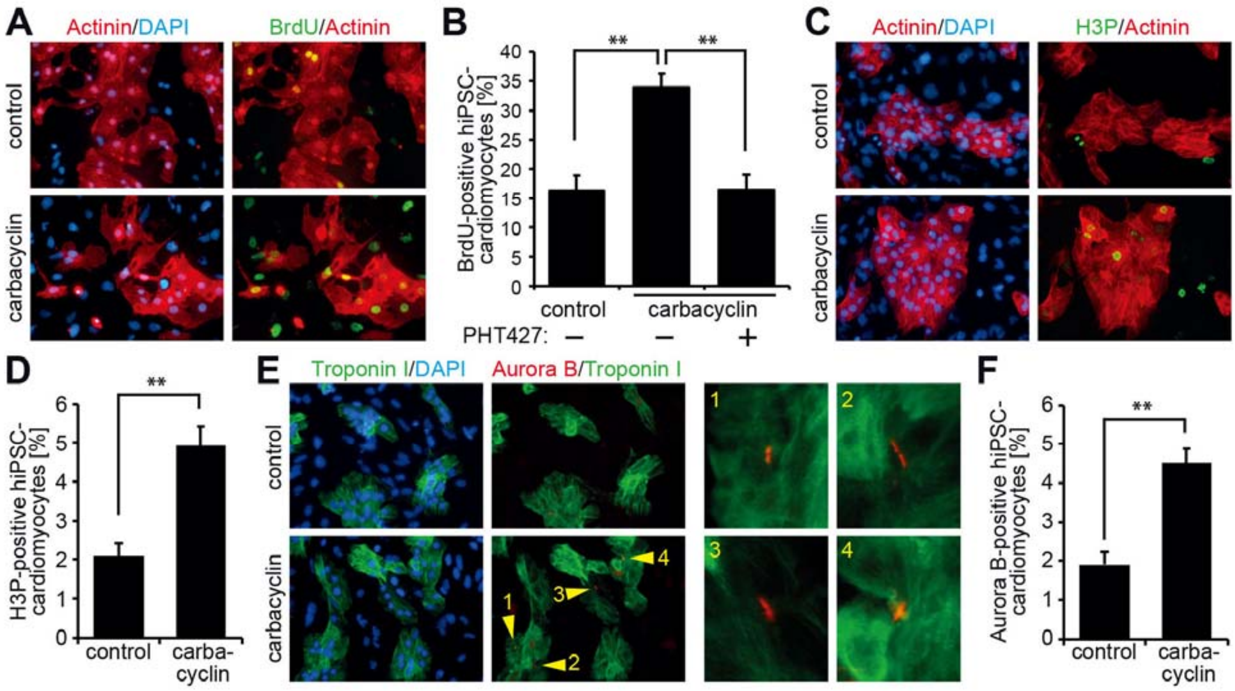

Supplementary information, Figure S5 Carbacyclin induces proliferation of hiPSC-derived cardiomyocytes via PDK1. **(A)** Representative immunofluorescence images of hiPSC-derived cardiomyocytes stained for Actinin (red), BrdU (green) and DNA (DAPI, blue) 48 hours after carbacyclin stimulation (n = 3). Cells were labeled with BrdU during the last 24 hours of carbacyclin stimulation. **(B)** Quantitative analyses of **B** including the effect of the dual inhibitor of PDK1/Akt PHT427. **(C-F)** Representative immunofluorescence images and quantitative analyses (n = 3) of hiPSC-derived cardiomyocytes stained for Actinin (red) and H3P (green) or Troponin I (green) and Aurora B (red). Nuclei were visualized by staining DNA with DAPI (blue). $^{**}P < 0.01$
