# Supplementary material for: Live cell screening platform identifies PPARδ as a regulator of cardiomyocyte proliferation and cardiac repair
Source: Cell Res. 2017 Jun 16;27(8):1002–19. doi: 10.1038/cr.2017.84 (PMC5539351; doi:10.1038/cr.2017.84)
Supplement: Supplementary information, Figure S6 — PPARδ activity is required for adult cardiomyocyte proliferation in regenerating zebrafish hearts. [file cr201784x6.pdf]

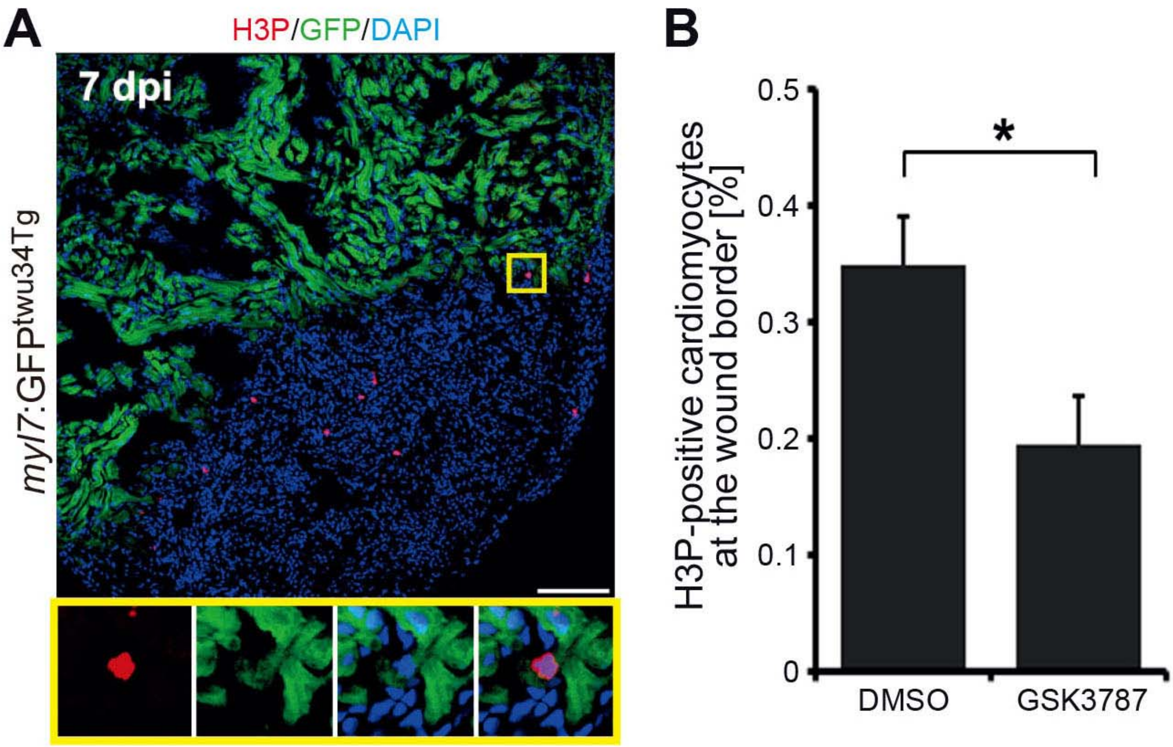

Supplementary information, Figure S6 PPARδ activity is required for adult cardiomyocyte proliferation in regenerating zebrafish hearts. **(A)** Representative images of cryoinjured *myl7*:GFP^{twu34TG} transgenic zebrafish showing GFP (green) in cardiomyocytes stained for H3P (red). Nuclei were visualized by staining DNA with DAPI (blue). **(B)** Quantitative analysis of H3P-positive cardiomyocytes (DMSO: n = 6 hearts; GSK3787: n = 7 hearts; $^*P <$ 0.05)
