# Supplementary material for: Live cell screening platform identifies PPARδ as a regulator of cardiomyocyte proliferation and cardiac repair
Source: Cell Res. 2017 Jun 16;27(8):1002–19. doi: 10.1038/cr.2017.84 (PMC5539351; doi:10.1038/cr.2017.84)
Supplement: Supplementary information, Figure S7 — Activation of caPPARδ does not induce an increase in c-kit+ cells in vivo after MI. [file cr201784x7.pdf]

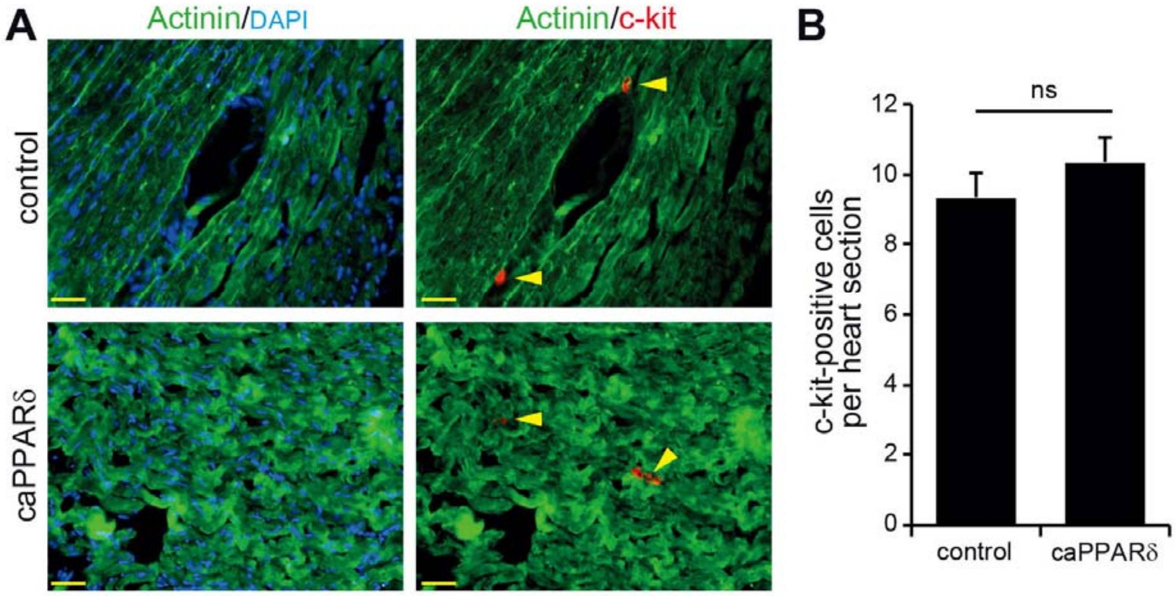


**Supplementary information, Figure S7** Activation of caPPARδ does not induce an increase in c-kit$^+$ cells *in vivo* after MI. Cardiomyocyte-restricted overexpression of a constitutively active PPARδ was induced in adult mice utilizing TMVPD mice via tamoxifen injections (caPPARδ). One week after tamoxifen injection, MI was induced via LAD ligation and hearts were analyzed two weeks later. **(A)** Representative heart sections from caPPARδ mice and control mice (TMCM) stained for c-kit, Actinin and nuclei (DAPI). **(B)** Quantitative analysis of c-kit$^+$ cells ($n$ = 4, **$P$ < 0.01). Scale bar = 25 μm. ns: not significant.
