# Supplementary material for: Live cell screening platform identifies PPARδ as a regulator of cardiomyocyte proliferation and cardiac repair
Source: Cell Res. 2017 Jun 16;27(8):1002–19. doi: 10.1038/cr.2017.84 (PMC5539351; doi:10.1038/cr.2017.84)
Supplement: Supplementary information, Figure S8 — PPARδ regulates Tbx20 expression at the transcriptional level. [file cr201784x8.pdf]

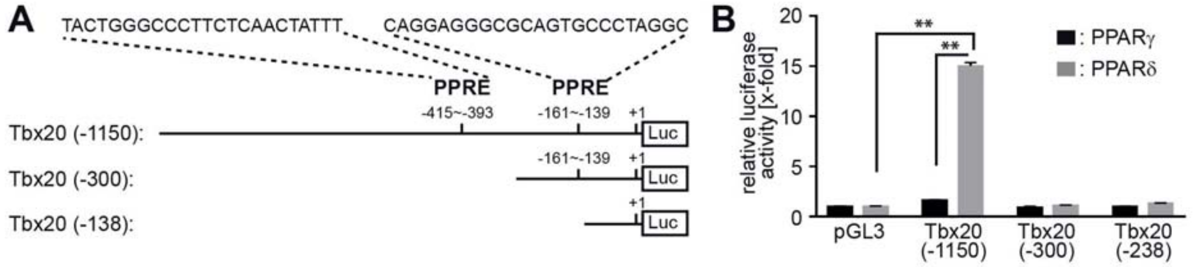

Supplementary information, Figure S8 PPARδ regulates Tbx20 expression at the transcriptional level. **(A)** Scheme of tested Tbx20 promoter reporter constructs. **(B)** PPARδ-induced transcriptional activation of a series of Tbx20 promoter reporter constructs in the pGL3-Basic plasmid for luciferase reporter assays. The Tbx20 promoter luciferase activities were normalized to renilla luciferase activities. $^{**}P < 0.01$. $n = 3$.
