# Supplementary material for: Live cell screening platform identifies PPARδ as a regulator of cardiomyocyte proliferation and cardiac repair
Source: Cell Res. 2017 Jun 16;27(8):1002–19. doi: 10.1038/cr.2017.84 (PMC5539351; doi:10.1038/cr.2017.84)
Supplement: Supplementary information, Figure S9 — Carbacyclin activates GSK3β/β-catenin signaling. [file cr201784x9.pdf]

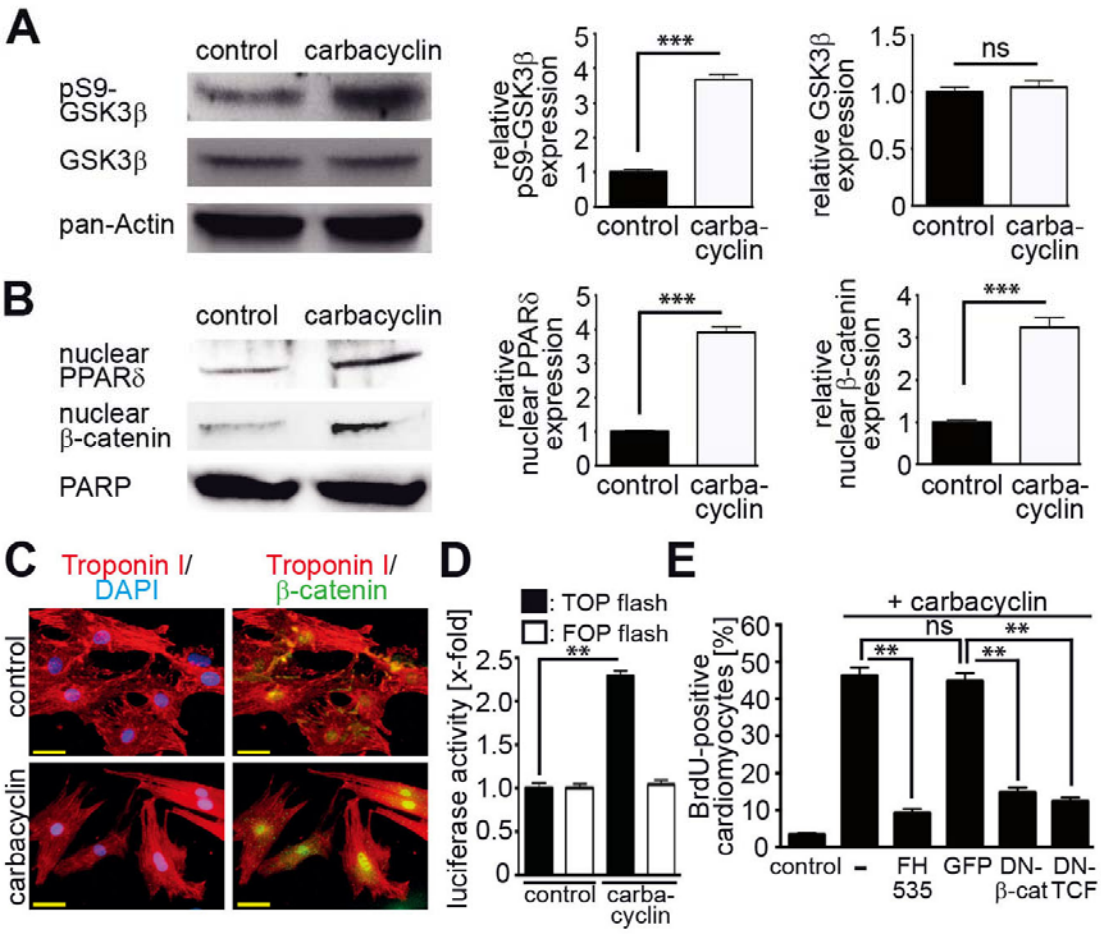

Supplementary information, Figure S9 Carbacyclin activates GSK3β/β-catenin signaling. **(A, B)** Representative examples of western blot analysis including densitometric quantification (n = 3) showing that carbacyclin increased **(A)** phosphorylation of glycogen synthase kinase 3 (pGSK3β) after 1h and **(B)** at 48 h nuclear expression of PPARδ and β-catenin. **(C)** Representative examples of neonatal cardiomyocytes (red, Troponin I) stained for β-catenin (green) showing nuclear accumulation of β-catenin upon carbacyclin treatment. **(D)** Carbacyclin induced β-catenin-mediated transcriptional activation, which was assayed by using reporter containing either functional (TOPflash) or mutated (FOPflash) Tcf binding sites. **(E)** β-catenin inhibition by adenoviral overexpression of dominant negative (DN) β-catenin or TCF as well as blocking the activity of β-catenin/PPARδ complexes by FH535 abolished the effect of carbacyclin on cardiomyocyte DNA synthesis (BrdU incorporation).). $**P < 0.01$. $***P < 0.001$. ns: not significant. Scale bar = 25 µm
