# Supplementary material for: Live cell screening platform identifies PPARδ as a regulator of cardiomyocyte proliferation and cardiac repair
Source: Cell Res. 2017 Jun 16;27(8):1002–19. doi: 10.1038/cr.2017.84 (PMC5539351; doi:10.1038/cr.2017.84)
Supplement: Supplementary information, Table S1 — Magadum et al. Table S1 [file cr201784x10.pdf]

Magadum *et al.* Table S1

Screening results

No.	compounds	time [h]	12	24	36	48	60	72	84	96
**1**	**DMSO**		**0**	**0**	**1**	**1**	**0**	**1**	**0**	**0**
2	25-Hydroxyvitamin D3	10 nm	1	1	1	1	1	0	0	0
		250 nm	1	1	1	1	0	1	0	0
		30 µM	1	1	1	1	0	0	0	0
**3**	**Retinoic acid, all trans**	**10 nm**	**1**	**2**	**2**	**2**	**2**	**2**	**1**	**1**
		250 nm	1	2	1	1	1	1	1	1
		30 µM	1	1	1	1	1	1	1	1
4	9-cis Retinoic acid	10 nm	1	2	2	1	1	1	0	0
		250 nm	1	1	1	1	1	1	1	1
		30 µM	1	1	1	1	0	0	0	0
5	13-cis Retinoic acid	10 nm	1	1	2	2	1	1	1	1
		250 nm	1	1	1	1	1	0	0	0
		30 µM	1	1	1	1	0	1	0	0
6	4-Hydroxyphenylretinamide	10 nm	1	1	1	1	1	1	0	0
		250 nm	0	1	1	1	1	1	0	0
		30 µM	1	1	1	1	0	0	0	0
7	AM-580	10 nm	1	1	1	1	1	1	1	1
		250 nm	1	1	1	0	1	0	0	0
		30 µM	1	1	1	1	0	0	0	0
**8**	**TTNPB**	**10 nm**	**2**	**2**	**2**	**3**	**2**	**1**	**1**	**1**
		250 nm	1	2	1	1	1	1	1	1
		30 µM	0	1	1	0	0	0	0	0
9	Methoprene acid	10 nm	1	1	1	1	1	1	1	1
		250 nm	1	1	1	1	0	1	1	1
		30 µM	1	1	1	1	0	0	0	0
10	WY-14643	10 nm	0	1	1	1	1	0	1	1
		250 nm	1	1	0	1	0	1	1	1
		30 µM	1	1	1	1	1	1	1	1
11	Ciglitazone	10 nm	1	0	1	0	1	1	0	0
		250 nm	1	1	1	1	0	0	0	0
		30 µM	0	1	1	0	0	0	0	0
12	Tetradecylthioacetic acid	10 nm	1	1	1	1	1	0	0	0
		250 nm	1	1	1	1	0	1	0	0
		30 µM	1	1	0	1	0	0	0	0
13	5,8,11,14-Eicosatetraynoic acid	10 nm	0	1	1	1	0	1	1	1
		250 nm	1	1	1	1	1	0	0	0
		30 µM	1	1	1	0	0	0	0	0
14	6-Formylindolo [3,2-B] carbazole	10 nm	1	1	1	1	1	0	0	0
		250 nm	1	1	1	1	0	0	0	0
		30 µM	1	1	1	0	0	0	0	0

15	Diindolylmethane	10 nm	1	1	1	0	0	0	0	0
		250 nm	1	1	0	0	0	0	0	0
		30 µM	0	0	0	0	0	0	0	0
16	Acetyl-S-farnesyl-L-cysteine	10 nm	1	1	1	1	1	1	1	1
		250 nm	1	1	1	1	1	0	0	0
		30 µM	1	1	0	0	0	1	0	0
17	S-Farnesyl-L-cysteine	10 nm	1	1	1	1	1	1	1	1
	methyl ester	250 nm	1	1	1	1	1	1	1	1
		30 µM	1	0	0	0	0	0	0	0
18	N-Acetyl-S-geranygeranyl-	10 nm	1	1	1	1	1	1	1	1
	L-cysteine	250 nm	1	1	1	1	1	1	1	1
		30 µM	1	1	1	1	1	1	1	1
19	AGC (Acetyl-geranyl-cysteine)	10 nm	1	1	1	1	0	1	0	0
		250 nm	1	1	1	0	0	0	0	0
		30 µM	0	1	1	1	0	0	0	0
20	Farnesylthioacetic acid	10 nm	1	1	1	1	0	1	0	0
		250 nm	1	1	1	1	0	1	0	0
		30 µM	1	0	0	0	0	0	0	0
21	Bezafibrate	10 nm	1	1	1	1	1	1	0	0
		250 nm	1	1	1	1	0	1	0	0
		30 µM	1	1	1	1	0	0	1	1
22	LY 171883	10 nm	1	1	1	1	1	0	0	0
		250 nm	1	1	1	1	1	0	0	0
		30 µM	1	1	1	1	0	0	0	0
23	15-Deoxy-D12,14-	10 nm	1	1	0	1	1	0	1	1
	prostaglandin J2	250 nm	1	0	1	1	0	0	0	0
		30 µM	1	1	1	1	1	0	0	0
24	Troglitazone	10 nm	1	1	1	1	1	1	1	1
		250 nm	1	1	1	1	0	0	0	0
		30 µM	0	1	0	0	0	0	0	0
25	CITCO	10 nm	1	1	1	1	1	1	1	1
		250 nm	1	1	1	0	0	0	0	0
		30 µM	1	1	0	0	0	0	0	0
26	Paxilline	10 nm	1	1	1	1	1	1	1	1
		250 nm	1	1	1	1	1	0	0	0
		30 µM	0	1	0	0	0	0	0	0
27	24(S)-Hydroxycholesterol	10 nm	1	1	0	1	0	1	0	0
		250 nm	1	1	1	1	0	0	0	0
		30 µM	0	1	0	0	0	0	0	0
28	24(S),25-Epoxycholesterol	10 nm	0	1	1	0	0	0	1	1
		250 nm	1	1	1	0	0	0	0	0
		30 µM	1	1	0	0	0	0	0	0
29	Pregnenolone-16(alpha)-	10 nm	1	1	1	0	1	0	0	0
	carbonitrile	250 nm	1	1	1	0	0	0	0	0

	30 µM	1	1	0	0	0	0	0	0
30 **Carbacyclin**	10 nm	1	2	2	2	2	1	1	1
	**250 nm**	**4**	**8**	**9**	**9**	**8**	**6**	**5**	**5**
	30 µM	1	1	1	1	1	1	1	1
31 Clofibric acid	10 nm	0	1	1	1	0	1	0	0
	250 nm	1	1	1	1	1	0	0	0
	30 µM	0	1	1	1	0	1	0	0
32 BADGE	10 nm	1	1	0	1	1	0	0	0
	250 nm	1	1	1	1	0	1	0	0
	30 µM	1	1	0	1	0	0	0	0
33 GW 9662	10 nm	1	1	2	1	1	1	1	1
	250 nm	1	1	0	0	0	0	0	0
	30 µM	1	0	0	0	0	0	0	0
34 Gemfibrozil	10 nm	0	1	1	1	1	0	1	1
	250 nm	1	1	1	1	1	0	0	0
	30 µM	1	0	0	0	0	1	0	0
35 GW 7647	10 nm	1	1	1	1	1	1	1	1
	250 nm	1	1	2	1	1	1	1	1
	30 µM	1	1	1	1	0	0	0	0
36 3,5-Diiodo-L-thyronine	10 nm	1	1	1	1	0	1	0	0
	250 nm	1	1	1	1	1	0	0	0
	30 µM	1	1	1	0	0	0	0	0
37 3,5-Diiodo-L-tyrosine	10 nm	0	1	1	1	1	1	0	0
	250 nm	1	1	1	1	0	0	1	1
	30 µM	1	1	1	0	0	0	0	0
38 all-trans-Retinol	10 nm	1	1	1	1	0	1	1	1
	250 nm	1	1	1	1	1	1	1	1
	30 µM	1	1	0	0	0	0	0	0
39 13-cis-Retinol	10 nm	1	1	1	1	1	1	1	1
	250 nm	1	1	1	1	0	0	0	0
	30 µM	1	1	0	0	0	0	0	0
40 Retinyl acetate	10 nm	1	1	1	1	1	1	1	1
	250 nm	1	2	2	1	1	0	0	0
	30 µM	1	0	0	0	0	0	0	0
41 3,5-Diiodo-4-hydroxy-	10 nm	1	1	1	1	1	1	0	0
phenylpropionic acid	250 nm	1	1	1	1	0	0	0	0
	30 µM	1	1	0	0	0	0	0	0
42 Cholic acid	10 nm	1	1	1	1	0	1	0	0
	250 nm	1	1	1	1	0	0	0	0
	30 µM	1	1	0	0	0	0	0	0
43 Deoxycholic acid	10 nm	0	1	1	1	1	1	1	1
	250 nm	1	1	1	1	1	0	1	1
	30 µM	1	0	1	1	0	0	0	0
44 Chenodeoxycholic acid	10 nm	1	1	1	1	0	0	0	0

		250 nm	0	0	0	0	0	0	0	0
		30 µM	0	0	0	0	0	0	0	0
45	Glycocholic acid	10 nm	1	1	1	0	0	0	1	1
		250 nm	1	0	0	0	0	0	0	0
		30 µM	0	0	0	0	0	0	0	0
46	Glycodeoxycholic acid	10 nm	1	0	0	0	0	0	0	0
		250 nm	0	0	0	0	0	0	0	0
		30 µM	1	1	1	1	1	1	1	1
47	Taurocholic acid	10 nm	1	1	1	1	0	0	0	0
		250 nm	1	0	0	0	0	0	0	0
		30 µM	0	0	0	0	0	0	0	0
48	Taurodeoxycholic acid	10 nm	1	1	1	1	1	0	0	0
		250 nm	1	1	1	1	0	0	0	0
		30 µM	1	1	0	0	0	0	0	0
49	Rifampicin	10 nm	1	1	1	1	1	1	0	0
		250 nm	1	1	1	1	1	1	0	0
		30 µM	1	1	1	1	1	0	0	0
50	Dexamethasone	10 nm	1	1	0	0	0	0	0	0
		250 nm	0	0	0	0	0	0	0	0
		30 µM	0	0	0	0	0	0	0	0
51	Lithocholic acid	10 nm	1	1	1	0	1	1	0	0
		250 nm	1	1	1	1	1	0	0	0
		30 µM	1	1	0	0	0	0	0	0
52	5b-Pregnan-3,20-dione	10 nm	1	1	1	1	1	0	1	1
		250 nm	1	1	2	1	1	0	0	0
		30 µM	1	0	0	0	0	0	0	0
53	Hyperforin	10 nm	1	1	1	1	1	0	0	0
		250 nm	1	1	1	0	0	0	0	0
		30 µM	1	0	0	0	0	0	0	0
54	Farnesol	10 nm	1	1	1	1	1	1	0	0
		250 nm	1	1	2	1	1	0	1	1
		30 µM	1	1	1	0	0	0	0	0
55	3a, 5a-Androstenol	10 nm	1	1	1	1	0	1	1	1
		250 nm	1	1	1	1	0	0	0	0
		30 µM	1	1	0	0	0	0	0	0
56	TCPOBOP	10 nm	1	1	1	1	1	0	0	0
		250 nm	1	1	1	1	0	0	0	0
		30 µM	1	0	0	0	0	0	0	0
57	N-Oleoylethanolamide	10 nm	1	1	1	1	1	1	0	0
		250 nm	1	1	1	1	0	0	0	0
		30 µM	1	0	0	0	0	0	0	0
58	GW4064	10 nm	1	1	1	1	1	0	0	0
		250 nm	1	1	1	1	1	0	0	0
		30 µM	1	1	1	0	0	0	0	0

59	Geranylgeraniol	10 nm	1	1	1	1	1	1	0	0
		250 nm	1	1	1	1	1	0	0	0
		30 µM	1	0	0	0	0	0	0	0
60	6a-Fluorotestosterone	10 nm	1	1	1	1	1	0	0	0
		250 nm	1	1	1	0	0	0	0	0
		30 µM	1	0	0	0	0	0	0	0
61	Tamoxifen	10 nm	1	1	1	1	1	1	0	0
		250 nm	1	1	1	1	1	0	0	0
		30 µM	0	0	0	0	0	0	0	0
62	Mifepristone	10 nm	1	1	1	1	0	0	0	0
		250 nm	1	1	1	0	0	0	0	0
		30 µM	0	0	0	0	0	0	0	0
63	Estrone	10 nm	1	1	1	1	1	1	0	0
		250 nm	1	1	1	0	0	0	0	0
		30 µM	0	0	0	0	0	0	0	0
64	13(S)-Hydroxy-9Z,	10 nm	1	1	1	1	1	0	0	0
	11E-octadecadienoic acid	250 nm	1	1	1	0	0	0	0	0
		30 µM	0	0	0	0	0	0	0	0
65	Cortisone	10 nm	1	1	1	1	1	0	0	0
		250 nm	1	0	1	0	0	0	0	0
		30 µM	0	0	0	0	0	0	0	0
66	Progesterone	10 nm	1	1	1	1	1	0	0	0
		250 nm	1	1	1	0	0	0	0	0
		30 µM	1	1	0	0	0	0	0	0
67	17b-Estradiol	10 nm	1	1	1	1	1	1	0	0
		250 nm	0	1	1	1	1	0	0	0
		30 µM	1	0	0	0	0	0	0	0
68	Pregnenolone	10 nm	0	1	1	1	1	0	0	0
		250 nm	1	1	1	1	0	0	0	0
		30 µM	1	0	0	0	0	0	0	0
69	Androstenedione	10 nm	1	1	1	1	1	0	0	0
		250 nm	1	1	1	1	0	0	0	0
		30 µM	1	0	0	0	0	0	0	0
70	1a,25-Dihydroxyvitamin D3	10 nm	0	1	1	1	1	1	1	0
		250 nm	1	1	1	1	0	0	0	0
		30 µM	1	0	0	0	0	0	0	0
71	Docosa-4Z,7Z,10Z,13Z,	10 nm	1	1	1	1	1	0	0	1
	16Z,19Z-hexaenoic acid	250 nm	1	1	1	1	0	0	0	0
		30 µM	1	0	0	0	0	0	0	0
72	3-Methylcholanthrene	10 nm	1	1	1	1	1	1	1	0
		250 nm	1	1	1	1	0	0	0	0
		30 µM	0	0	0	0	0	0	0	0
73	Acitretin	10 nm	1	1	1	1	1	0	0	1
		250 nm	1	1	1	1	0	0	0	0

	30 µM	1	0	0	0	0	0	0	0
74 Pioglitazone	10 nm	1	1	1	1	1	0	0	0
	250 nm	1	1	1	1	1	0	0	0
	30 µM	1	0	0	0	0	0	0	0
75 **4-Hydroxyretinoic acid**	**10 nm**	**2**	**3**	**3**	**2**	**2**	**1**	**0**	**0**
	250 nm	1	1	1	1	1	1	1	0
	30 µM	1	1	1	0	0	0	0	0
76 3-amino benzamide	10 nm	1	1	0	1	1	1	1	0
	250 nm	1	0	0	1	1	1	1	1
	30 µM	1	0	1	0	0	0	0	0
77 Phthalazinone pyrazole	10 nm	1	1	1	1	1	1	1	1
	250 nm	1	0	1	0	0	0	0	1
	30 µM	1	1	1	1	0	0	0	0
78 AGK2	10 nm	1	1	1	0	1	1	0	1
	250 nm	1	1	1	1	1	1	1	0
	30 µM	1	1	1	0	0	0	0	0
79 Mirin	10 nm	1	1	1	1	1	0	0	0
	250 nm	1	1	1	1	0	0	1	1
	30 µM	1	1	1	1	1	0	0	0
80 chidamide	10 nm	1	1	1	1	1	1	0	0
	250 nm	1	1	1	1	1	1	1	1
	30 µM	1	1	1	1	0	0	0	0
81 SAHA	10 nm	1	1	1	1	1	1	1	0
	250 nm	1	1	1	1	0	0	1	1
	30 µM	1	1	0	1	0	0	0	0
82 F-Amidine	10 nm	1	1	1	1	1	0	1	1
	250 nm	1	1	1	1	0	1	0	1
	30 µM	1	1	0	0	0	0	0	0
83 4-iodo-SAHA	10 nm	1	1	1	1	1	0	1	1
	250 nm	1	1	1	1	1	0	0	0
	30 µM	1	1	0	0	0	0	0	0
84 UNC0321	10 nm	1	1	1	1	0	0	0	1
	250 nm	1	1	0	0	1	0	0	0
	30 µM	1	0	0	0	0	0	0	0
85 Isoliquiritigenin	10 nm	1	1	1	1	0	1	1	0
	250 nm	1	1	1	1	1	0	0	0
	30 µM	1	0	0	0	0	0	0	0
86 CAY10603	10 nm	1	0	1	1	0	0	1	1
	250 nm	1	1	1	0	0	0	0	0
	30 µM	1	1	1	0	0	0	0	0
87 Pimelic diphenylamide 106	10 nm	1	1	1	1	0	1	0	1
	250 nm	1	1	1	1	1	0	0	0
	30 µM	1	1	1	0	0	0	0	0
88 3-deazaneplanocin-A	10 nm	1	1	1	1	1	0	1	0

		250 nm	1	1	1	1	0	0	0	0
		30 µM	1	1	0	0	0	0	0	0
89	2,4-OPD	10 nm	1	1	1	1	1	0	0	1
		250 nm	1	1	1	1	1	1	1	0
		30 µM	1	0	1	1	0	0	0	0
90	2-PCPA (hydrochloride)	10 nm	1	1	1	1	1	1	1	0
		250 nm	1	1	1	1	0	1	0	1
		30 µM	1	1	0	0	0	0	0	0
91	Tubastatin A (trifluroacetate salt)	10 nm	1	1	1	1	1	0	0	1
		250 nm	1	1	0	0	0	0	0	0
		30 µM	1	0	1	0	0	0	0	0
92	**Nicotinamide**	10 nm	1	1	1	1	1	1	1	0
		**250 nm**	**1**	**2**	**2**	**2**	**2**	**2**	**1**	**0**
		30 µM	1	1	1	0	1	0	0	0
93	Zebularine	10 nm	1	1	1	1	0	1	0	1
		250 nm	1	1	1	1	0	1	1	1
		30 µM	1	1	0	0	0	0	0	0
94	(S)-HDAC-42	10 nm	1	1	1	1	1	1	1	0
		250 nm	1	1	1	1	1	1	1	1
		30 µM	1	1	1	1	1	1	1	0
95	Trans-resveratrol	10 nm	1	1	1	1	1	0	0	1
		250 nm	1	1	1	1	1	0	1	1
		30 µM	1	1	1	0	0	0	0	1
96	DMOG	10 nm	1	1	1	1	0	1	1	0
		250 nm	1	1	1	1	1	1	0	1
		30 µM	1	1	1	1	1	0	1	0
97	Cl-Amidine	10 nm	1	1	1	1	1	1	0	1
		250 nm	1	1	1	1	0	0	1	0
		30 µM	1	1	1	0	0	0	0	1
98	Garcinol	10 nm	1	1	1	1	0	1	0	0
		250 nm	1	1	1	1	1	1	1	1
		30 µM	1	1	1	0	0	0	0	0
99	BIX01284 (hydrocholride hydrate)	10 nm	1	1	1	1	1	1	0	0
		250 nm	1	1	1	1	1	0	0	1
		30 µM	1	1	1	1	0	0	0	0
100	Valproic acid (sodium salt)	10 nm	1	1	1	1	1	0	1	0
		250 nm	1	1	1	1	1	1	1	0
		30 µM	1	1	1	1	1	0	1	0
101	splitomicin	10 nm	1	1	1	1	1	0	1	1
		250 nm	1	1	1	1	1	1	0	1
		30 µM	1	1	1	1	0	1	0	1
102	MS-275	10 nm	1	1	1	1	1	1	0	1
		250 nm	1	1	1	1	0	0	1	0
		30 µM	1	1	1	0	1	0	0	0

103 trichostatin A	10 nm	0	1	1	1	1	1	1	0
	250 nm	1	1	1	1	0	0	0	1
	30 µM	1	0	1	0	0	0	0	0
104 Ellagic acid	10 nm	1	1	1	1	0	1	0	1
	250 nm	1	1	1	1	0	0	0	0
	30 µM	1	1	1	1	1	0	0	0
105 Suramin (sodium salt)	10 nm	1	1	1	0	1	1	1	0
	250 nm	1	1	1	1	1	0	0	0
	30 µM	1	1	1	1	0	0	0	0
106 Tenovin-1	10 nm	1	1	1	1	1	0	0	1
	250 nm	1	1	1	1	0	1	0	0
	30 µM	1	1	1	0	0	0	0	0
107 CBHA	10 nm	1	1	1	1	1	0	1	0
	250 nm	1	1	1	1	1	0	0	0
	30 µM	1	1	1	1	0	0	0	0
108 HNHA	10 nm	1	1	1	1	1	1	1	1
	250 nm	1	1	1	1	1	1	1	0
	30 µM	1	1	1	1	1	1	1	0
109 (-)- Neplanocin A	10 nm	1	1	1	1	1	0	1	1
	250 nm	1	1	1	1	1	0	0	1
	30 µM	1	1	1	1	0	0	0	1
110 Sciptaid	10 nm	1	1	1	1	1	1	0	1
	250 nm	1	1	1	1	0	0	1	0
	30 µM	1	1	1	1	0	0	0	0
111 JGB1741	10 nm	1	1	1	1	0	1	1	0
	250 nm	1	1	1	1	1	0	0	1
	30 µM	1	1	1	1	1	1	0	0
112 Tinovin-6	10 nm	0	1	1	1	1	1	1	1
	250 nm	1	1	1	1	1	1	1	0
	30 µM	1	1	1	1	0	0	0	0
113 M-344	10 nm	1	1	1	1	0	1	0	1
	250 nm	1	1	1	1	0	1	0	1
	30 µM	1	1	1	0	0	0	0	0
**114 RG-108**	10 nm	1	1	1	1	1	1	0	0
	**250 nm**	**1**	**2**	**2**	**3**	**3**	**2**	**1**	**0**
	30 µM	1	1	1	1	1	1	1	0
**115 CAY10433**	10 nm	1	1	2	1	1	1	1	0
	**250 nm**	**2**	**2**	**3**	**3**	**3**	**2**	**1**	**1**
	30 µM	1	1	1	1	1	1	0	1
**116 Sinefungin**	10 nm	1	1	1	1	1	0	1	1
	**250 nm**	**1**	**2**	**2**	**2**	**2**	**1**	**1**	**1**
	30 µM	1	1	1	1	0	1	0	0
117 Suberohydroxamic acid	10 nm	1	1	1	1	1	0	1	1
	250 nm	1	1	1	1	0	0	0	1

		30 µM	1	0	0	0	0	0	0	0
118	I-BET	10 nm	1	1	1	1	1	1	0	1
		250 nm	0	1	1	1	1	0	0	0
		30 µM	1	1	1	1	0	0	0	0
119	Sodium butyrate	10 nm	1	1	1	1	1	0	0	0
		250 nm	1	1	1	1	0	0	0	0
		30 µM	1	1	1	0	0	0	0	0
120	Oxamflatin	10 nm	1	1	1	1	1	0	0	0
		250 nm	1	0	0	0	0	0	0	0
		30 µM	0	0	0	0	0	0	0	0
121	2,3,5-triacetyle-5-azacytidine	10 nm	1	1	1	1	0	1	0	0
		250 nm	1	1	1	1	1	0	0	0
		30 µM	1	1	1	0	0	0	0	0
122	piceatannol	10 nm	1	1	1	1	1	1	0	0
		250 nm	1	1	1	1	1	0	0	0
		30 µM	1	1	1	0	0	0	0	0
123	S-adenosylhomocysteine	10 nm	1	1	1	1	1	0	0	0
		250 nm	1	1	1	1	0	0	0	0
		30 µM	1	1	1	0	0	0	0	0
124	UNC0638	10 nm	1	1	1	1	1	0	1	0
		250 nm	0	1	1	1	0	1	0	0
		30 µM	1	1	1	1	0	0	0	0
125	Anacardic acid	10 nm	1	1	1	1	0	1	0	1
		250 nm	0	1	1	1	1	0	0	0
		30 µM	1	1	1	0	0	0	0	0
126	Salermide	10 nm	1	1	1	1	1	0	1	0
		250 nm	1	1	0	1	0	1	0	0
		30 µM	0	1	1	0	0	0	0	0
127	UNC0224	10 nm	1	1	1	0	0	1	0	1
		250 nm	1	1	1	0	0	0	0	0
		30 µM	1	1	0	0	0	1	0	0
128	EX-527	10 nm	1	1	1	1	1	1	1	0
		250 nm	1	1	1	1	0	1	1	0
		30 µM	1	1	0	1	1	1	1	0
129	CCG-100402	10 nm	1	1	1	1	1	0	0	1
		250 nm	1	1	1	1	0	0	1	1
		30 µM	1	1	1	0	0	0	0	1
**130**	**10% FCS**		**2**	**3**	**4**	**5**	**6**	**6**	**4**	**2**
**131**	**FGF1+ p38 inhibitor**		**3**	**8**	**9**	**10**	**10**	**9**	**7**	**3**

orange	controls
red	positive hits
