# Supplementary material for: Live cell screening platform identifies PPARδ as a regulator of cardiomyocyte proliferation and cardiac repair
Source: Cell Res. 2017 Jun 16;27(8):1002–19. doi: 10.1038/cr.2017.84 (PMC5539351; doi:10.1038/cr.2017.84)
Supplement: Supplementary information, Table S2 — Magadum et al. Table S2 [file cr201784x11.pdf]

Magadum *et al.* Table S2

**Parameters**	**control (TMCM)**	**caPPARδ (TMVPD)**
Heart Rate (BPM)	411.5 ± 9.63	436.7 ± 11.1
Endocardial Area; d (mm^2)	21.83 ± 0.82	23.3 ± 0.95
Endocardial Area; s (mm^2)	17.12 ± 0.90	16.69 ± 1.01
Endocardial Major; d (mm)	7.37 ± 0.16	7.33 ± 0.05
Endocardial Major; s (mm)	7.14 ± 0.17	6.70 ± 0.09*
Epicardial Area; d (mm^2)	35.17 ± 0.86	36.44 ± 0.96
Epicardial Area; s (mm^2)	30.61 ± 1.11	30.30 ± 0.98
Epicardial Major; d (mm)	8.16 ± 0.13	8.04 ± 0.07
Epicardial Major; s (mm)	7.93 ± 0.15	7.53 ± 0.08*
Endocardial Volume; d (µl)	55.21 ± 3.42	63.43 ± 4.80
Endocardial Volume; s (µl)	35.25 ± 3.11	35.97 ± 3.88
Endocardial Stroke Volume (µl)	19.97 ± 2.42	27.47 ± 2.55*
Endocardial EF (%)	36.31 ± 3.66	43.75 ± 3.63
Endocardial FAC (%)	21.59 ± 2.67	28.56 ± 2.49*
Endocardial Area Change (mm^2)	4.70 ± 0.63	6.61 ± 0.54*
Endocardial CO (ml/min)	8.18 ± 1.02	11.93 ± 1.94*

*: indicates a p-value of $p < 0.05$
