# Supplementary material for: Live cell screening platform identifies PPARδ as a regulator of cardiomyocyte proliferation and cardiac repair
Source: Cell Res. 2017 Jun 16;27(8):1002–19. doi: 10.1038/cr.2017.84 (PMC5539351; doi:10.1038/cr.2017.84)
Supplement: Supplementary information, Table S3 — Magadum et al. Table S3 [file cr201784x12.pdf]

Magadum *et al.* Table S3

**Parameters (long-term)**	**control (TMCM)**	**caPPARδ (TMVPD)**
Heart Rate (BPM)	375±18.2	386±30.5
Endocardial Area; d (mm^2)	28.74±1.46	25.81±1.49
Endocardial Area; s (mm^2)	23.68±1.32	19.95±1.08*
Endocardial Major; d (mm)	7.82±0.14	7.42±0.18
Endocardial Major; s (mm)	7.50±0.13	7.14±0.17
Epicardial Area; d (mm^2)	46.26±1.7	41.72±1.69
Epicardial Area; s (mm^2)	41.38±1.9	37.79±1.41
Epicardial Major; d (mm)	8.77±0.15	8.46±0.16
Epicardial Major; s (mm)	8.46±0.12	8.19±0.14
Endocardial Volume; d (µl)	90.55±7.79	76.84±7.27
Endocardial Volume; s (µl)	64.38±6.41	47.66±4.18*
Endocardial Stroke Volume (µl)	26.16±2.52	29.18±3.16
Endocardial EF (%)	29.23±1.92	37.68±0.85*
Endocardial FAC (%)	17.65±1.31	22.53±0.97*
Endocardial Area Change (mm^2)	5.06±0.44	5.86±0.49
Endocardial CO (ml/min)	9.95±1.31	11.67±2.24

*: indicates a p-value of $p < 0.05$
