# Supplementary material for: Live cell screening platform identifies PPARδ as a regulator of cardiomyocyte proliferation and cardiac repair
Source: Cell Res. 2017 Jun 16;27(8):1002–19. doi: 10.1038/cr.2017.84 (PMC5539351; doi:10.1038/cr.2017.84)
Supplement: Supplementary information, Table S4 — Magadum et al. Table S4 [file cr201784x13.pdf]

Magadum *et al.* Table S4

Parameters	DMSO		GW0742	
	TMCM	PPARδ+/-	TMCM	PPARδ+/-
Heart Rate (BPM)	372.3 ± 13.2	410.2 ± 28.53	401.0 ± 14.3	380.2 ± 13.6
Endocardial Area; d (mm^2)	27.71 ± 1.43	28.26 ± 1.43	26.21 ± 1.20	26.20 ± 1.15
Endocardial Area; s (mm^2)	21.38 ± 1.10	22.79 ± 1.31	19.27 ± 1.23	20.54 ± 0.98
Endocardial Major; d (mm)	7.79 ± 0.22	8.04 ± 0.23	7.80 ± 0.28	7.66 ± 0.21
Endocardial Major; s (mm)	7.35 ± 0.23	7.72 ± 0.21	7.18 ± 0.30	7.29 ± 0.20
Epicardial Area; d (mm^2)	42.74 ± 1.41	43.66 ± 1.60	41.04 ± 1.63	40.48 ± 1.30
Epicardial Area; s (mm^2)	36.89 ± 1.34	39.25 ± 1.82	35.41 ± 1.93	35.44 ± 1.29
Epicardial Major; d (mm)	8.60 ± 0.20	8.85 ± 0.21	8.53 ± 0.27	8.52 ± 0.18
Epicardial Major; s (mm)	8.20 ± 0.20	8.60 ± 0.19	8.10 ± 0.28	8.18 ± 0.17
Endocardial Volume; d (µl)	84.07 ± 6.58	84.74 ± 6.32	75.05 ± 4.80	76.49 ± 5.12
Endocardial Volume; s (µl)	53.02 ± 3.93	57.60 ± 5.38	44.22 ± 4.17	49.58 ± 3.80
Endocardial Stroke Volume (µl)	31.05 ± 2.76	27.14 ± 2.11	30.83 ± 1.60	26.91 ± 1.86
Endocardial EF (%)	36.81 ± 0.74	32.34 ± 2.20	41.72 ± 2.06*	35.38 ± 1.55*
Endocardial FAC (%)	22.85 ± 0.49	19.41 ± 1.59*	26.82 ± 1.72*	21.64 ± 1.29*
Endocardial Area Change (mm^2)	6.34 ± 0.37	5.47 ± 0.47	6.94 ± 0.40	5.66 ± 0.39*
Endocardial CO (ml/min)	11.63 ± 1.24	10.99 ± 1.19	12.45 ± 0.96	10.31 ± 0.91

*: indicates a p-value of $p < 0.05$ (PPARδ+/- vs. TMCM or DMSO vs. GW0742 for the TMCM group)
